# Retroviral integrations contribute to elevated host cancer rates during germline invasion

**DOI:** 10.1038/s41467-021-21612-7

**Published:** 2021-02-26

**Authors:** Gayle K. McEwen, David E. Alquezar-Planas, Anisha Dayaram, Amber Gillett, Rachael Tarlinton, Nigel Mongan, Keith J. Chappell, Joerg Henning, Milton Tan, Peter Timms, Paul R. Young, Alfred L. Roca, Alex D. Greenwood

**Affiliations:** 1 Department of Wildlife Diseases, Leibniz Institute for Zoo and Wildlife Research, Berlin, Germany; 2 Australian Museum Research Institute, Australian Museum, Sydney, NSW Australia; 3 Institute for Neurophysiology, Charité-Universitätsmedizin Berlin, Berlin, Germany; 4 Australia Zoo Wildlife Hospital, Beerwah, QLD Australia; 5 Faculty of Medicine and Health Sciences, University of Nottingham, Leicestershire, UK; 6 School of Chemistry & Molecular Biosciences, University of Queensland, Brisbane, QLD Australia; 7 School of Veterinary Science, University of Queensland, Brisbane, QLD Australia; 8 Illinois Natural History Survey, University of Illinois at Urbana–Champaign, Champaign, IL USA; 9 Genecology Research Center, University of the Sunshine Coast, Sippy Downs, QLD Australia; 10 Department of Animal Sciences, University of Illinois at Urbana–Champaign, Urbana, IL USA; 11 Department of Veterinary Medicine, Freie Universität, Berlin, Germany

**Keywords:** Cancer genetics, Cancer genomics, Evolutionary biology, Retrovirus

## Abstract

Repeated retroviral infections of vertebrate germlines have made endogenous retroviruses ubiquitous features of mammalian genomes. However, millions of years of evolution obscure many of the immediate repercussions of retroviral endogenisation on host health. Here we examine retroviral endogenisation during its earliest stages in the koala (*Phascolarctos cinereus*), a species undergoing germline invasion by koala retrovirus (KoRV) and affected by high cancer prevalence. We characterise KoRV integration sites (IS) in tumour and healthy tissues from 10 koalas, detecting 1002 unique IS, with hotspots of integration occurring in the vicinity of known cancer genes. We find that tumours accumulate novel IS, with proximate genes over-represented for cancer associations. We detect dysregulation of genes containing IS and identify a highly-expressed transduced oncogene. Our data provide insights into the tremendous mutational load suffered by the host during active retroviral germline invasion, a process repeatedly experienced and overcome during the evolution of vertebrate lineages.

## Abstract (web summary)

Koalas are susceptible to neoplasms, which are related to infection with the Koala retrovirus. Here, the authors use DNA sequencing to show that the retroviral insertion sites cluster near known cancer genes and demonstrate a high mutational load associated with the germline invasion of the virus.

## Introduction

Retrovirus-like elements are abundant in vertebrate genomes^1^, comprising 8% of the human genome. They originate when exogenous retroviruses infect the host germline and are subsequently inherited by offspring as endogenous retroviruses (ERVs), some of which eventually reach fixation. Since most mammalian ERVs derive from germline infections that occurred millions of years ago, the early and most critical stages of host–pathogen accommodation, including the immediate effects on host health, remain unclear. Retroviruses are insertional mutagens, affecting the host in multiple potentially deleterious ways, including the disruption or alteration of gene expression^2^. This makes the transition from a newly endogenised, active retrovirus to a genomically fixed ERV a complex and dynamic evolutionary process. The ongoing conflict between virus and host (sometimes termed a retroviral “invasion” of the host germline), unfolds over millions of years, during which time proviruses can frequently reintegrate in the host genome causing further germline mutations (de facto natural mutagenesis). The proviruses will also undergo mutations which can ultimately render them inactive.

The negative effects of exogenous retroviral infection of somatic cells have been well characterised^3,4^. In addition, the consequences of retroviral germline infection, where proviruses in the germline (particularly gammaretroviruses) are subject to Mendelian inheritance and are potentially active in every cell of an individual, have been extensively studied in inbred and wild-derived laboratory mouse strains^5^. Indeed much of the variation between laboratory mouse strains is thought to be due to variation in the number and location of ERVs they possess^5^. Examples of interactions of ERVs with their host’s genome include the accumulation of new germline insertions^6^, the production of active virus infections triggering oncogenesis^7^ and in some cases recombination of defective ERVs to create new infectious viruses^8^.

Given the mutagenic nature of retroviruses, a general consequence of ongoing germline invasion is predicted to be increased host cancer rates but this has not yet been directly observed in outbred wild species^7,8^. Due to their oncogenic potential, it is likely that intact ERVs will be strongly selected against in favour of defective versions^5^. Evidence for such selection has recently been reported which shows that ERVs can be rapidly degraded through recombination during the early stages of germline invasion^9^. The host population will experience a greatly increased mutational burden overall until all proviral copies are attenuated.

The koala (*Phascolarctos cinereus*) provides a means of studying the early stages of retroviral endogenisation in a wild, outbred, large-bodied mammal. Koalas are currently undergoing active germline invasion, a process which likely began in the last 50,000 years^10^. This recent germline infection is in contrast to other species with unfixed ERVs, such as mice where the most recent ERV group (murine leukaemia virus, MLV) entered the genome ca. 1.5 million years ago^11^. The negative health effects on koalas caused by this invasion are also of interest and concern from a conservation perspective for this iconic and vulnerable species. The koala retrovirus (KoRV-A)^12^ is endogenous in all koalas from northern Australia, but some southern koalas are believed to remain unaffected as the virus has spread from north to south^13^. Other KoRV variants (designated KoRV-B through KoRV-I)^14–16^ have also been detected but are presumed to be exogenous (infecting only somatic cells and transmitted horizontally). Endogenous KoRV-A may also produce infectious virus particles and be spread horizontally as well as vertically^13,17^, though the evidence is correlative, and blocking of the receptor^18^ by endogenous KoRV-A may limit exogenous retroviral spread if it does occur in vivo.

KoRV is a member of a retroviral lineage associated with lymphoma and leukaemia^19^, both of which occur with remarkably high prevalence in koalas. In the wild, nearly 3% of mature northern koalas are affected by lymphoma^20,21^ (which is at least an order of magnitude higher than in humans) and over 7% suffer from lymphoid neoplasms^21^. In a study of captive koalas, 25% developed lymphoma, with a potential hereditary pattern of disease observed in some facilities^22^. Lymphoid neoplasms are associated with high KoRV viral loads and are seen at a higher rate in northern populations^21,23^. Many other benign and malignant tumours are also observed such as osteochondromas, which affect 2–4.5% of northern koalas^20–22^, mainly occurring in the skull and resulting in death through compression of organs.

Here, we show that northern koalas harbour ca. 80–100 inherited proviral copies in their germlines, none of which are fixed across the population and few of which are shared between individuals. Shared integration sites (IS) correspond to the geographical proximity of the koalas, and also suggest a possible KoRV-mediated predisposition to tumours. We find that tumour tissues accumulate somatic KoRV integrations which are significantly associated with genes involved in cancer. In one koala, we detect a KoRV provirus that has transduced a host oncogene, with the oncogene displaying markedly increased expression. Finally, we find genomic hotspots of KoRV integration in proximity to genes associated with cancer.

## Results

### Detection of KoRV IS in healthy and tumour tissue

We characterised KoRV IS in both tumour and healthy tissues from ten koalas from northern Australia. Details of the koalas, their diagnoses and the tissues used are provided in Supplementary Table 1. IS detection was carried out using a technique involving DNA sonication, inverse PCR (iPCR) and subsequent PacBio sequencing which we term SIP (for sonication iPCR)^24^. This generated long sequence reads (Supplementary Table 2) which span the regions of the koala genome flanking each IS and the adjoining proviral sequences (Supplementary Fig. 1). Mapping the reads to the koala genome (release version 4.1) allowed us to precisely identify the genomic position of each IS (Supplementary Fig. 2). A total of 1002 distinct IS were detected in the ten koalas (a full list is provided in Supplementary Data 1). Studies of KoRV have been facilitated by assembly of the koala genome^25^. Other more ancient ERVs are reported as rare in the koala genome (~1% of the genome) compared to other mammals, e.g. human, gorilla and mouse, where 8–12% of their genomes are composed of ancient ERVs. In general, Australian marsupials and monotremes are reported to contain a very low percentage of ERVs^25^, likely due to their geographical separation from species that act as retroviral reservoirs.

**Fig. 1:**
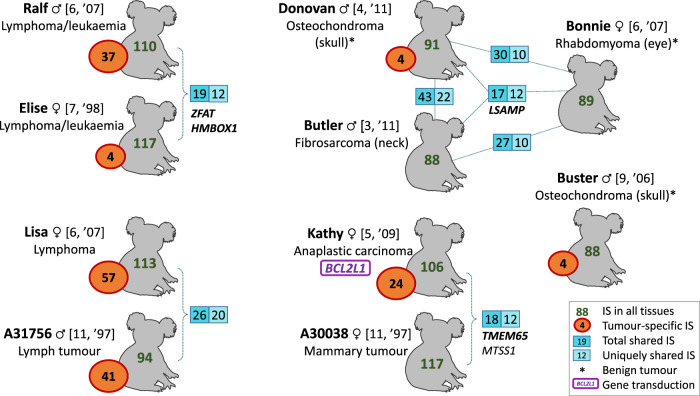
Overview of IS detected in healthy and tumour tissues of ten koalas. The name and sex of each koala is given, with age and year of birth in square brackets (age and year of birth are estimated from tooth wear). Number of IS detected in all tissues are shown in green for each koala and represent ERVs, whereas tumour-specific IS (shown in orange circles) represent somatic integrations in tumour tissues. Koalas sharing many IS are grouped (see Fig. 3), with the numbers of shared IS given in blue boxes (dark blue = total IS shared, including IS shared with other koalas; light blue = IS shared only between given koalas; lines connect koalas that share IS and do not represent specific family relationships). Outside of these groups, koalas share nine or fewer IS. Examples of genes containing IS shared only between indicated koalas are given (with genes containing IS in exons shown in boldface).

In each koala, the majority of IS were common to both healthy and tumour tissues (overall, 786 of the 1002 IS) (Fig. 1, Supplementary Table 3). The fact that most IS were independently identified in both healthy and tumour tissues indicates that IS detection using our SIP method^24^ was close to saturation. Moreover, all shared IS were found in both tissues of all koalas that shared the IS (if saturation had not been reached, IS would be missing in some tissues). Overall, false positives and negatives are unlikely to impact our findings (see Supplementary Figs. 1–3 for further details).

ERVs are present in all cells of an individual, being inherited in a Mendelian fashion. We found, on average, 100 KoRV ERVs per koala. This is in line with the number of KoRV proviruses found in the koala reference genome which has 98 KoRV ERVs, 65 of which are intact, full-length KoRV-A sequences^26^. Since the reference genome contains 65 intact copies of KoRV-A and KoRV genes are expressed in koalas^27^, it is plausible that some KoRV proviruses retain the potential to reintegrate (through reinfection or possibly retrotransposition), leading to further germline and/or somatic integrations. Therefore, in each of our koalas, some KoRV ERVs are likely to retain the ability to reintegrate and could potentially affect host gene expression. The majority of KoRV ERVs (79%) were not shared among the koalas examined. The lack of sharing of IS is reminiscent of inbred lab mouse strains where the formation of novel IS can occur, though at a very much lower rate than that seen in koalas^28^, but contrasts with older ERVs of vertebrates including those in humans which are generally found in all individuals at the same respective genomic loci. In koalas, the lack of fixed IS and the limited number of IS shared between individuals reflects the early stage of host germline invasion by KoRV and indicates a rapid expansion and accumulation of new IS across the northern koala population. It is not yet known whether new KoRV-A integrations are the result of (i) retroviral integration of a virus produced by an ERV in an individual; (ii) intracellular retrotransposition of an ERV; or (iii) new retroviral infection of an exogenous KoRV-A virus. However, in mice, active ERVs have been shown to produce infectious viruses from birth and generate new proviruses by infecting the oocytes of offspring in utero^6^.

KoRV-A and other KoRV variants were identified in our samples (Supplementary Table 4), with KoRV-A being by far the predominant subtype. Other subtypes are thought to be exogenous and thus present in a small proportion of host cells, so that KoRV-B, KoRV-D and KoRV-F represent only a tiny fraction of reads detected in any sample^29^. KoRV-B, which has been associated with cancer in a US zoo^15^, was found in only two of the koalas (Kathy and Butler) and therefore does not explain most of the observed neoplasms. Moreover, this variant of KoRV-B lacked the expanded repeats in the LTRs and the CETTG motif (shown to be more pathogenic than the CETAG/CGTAG motif present in KoRV-A) found in the zoo koalas. In this study, we have used the term KoRV to represent all insertions, recognising that nearly all IS in northern koalas are KoRV-A derived integrations, with a small number potentially arising from other KoRV variants.

### Tumour-specific IS show an association with cancer genes

Of the 1002 IS characterised, 172 IS were detected exclusively in tumour tissues (“tumour-unique IS”) (Fig. 1). These are somatic integrations that are the result of retroviral genomic reintegration followed by clonal expansion of the tumour cells. The presence of tumour-specific IS was not attributable to sequence coverage differences (Supplementary Fig. 3), and the tumour specificity of a subset of these IS was verified by PCR (Supplementary Fig. 4). A further 37 IS were detected exclusively in healthy tissues which represent other non-cancer-related somatic reintegration events (14 of these occurred in a single koala, Kathy) (Supplementary Table 3).

Four koalas with malignant neoplasms showed an accumulation of IS in tumours, with over 20 tumour-specific IS each. Tumour-specific IS were found more often than expected in exons (Supplementary Fig. 5), although these were mainly non-coding exons (Supplementary Table 5), including the 5′ UTRs of cancer-related genes *BCL2*, *IL6R*, *KRT13* and *TRAF4*. To investigate possible retroviral-mediated dysregulation of host genes, we selected the gene closest to each IS as a way to generate an unbiased gene set. Just under half (45.5%) of the 1002 IS are located within genes, and of the remainder, 74.5% are within 1 kb of a gene and 91% are within 50 kb. In each case, we determined the closest host gene, regardless of orientation. Although genes close to IS are the most likely to affected by retroviral-mediated dysregulation, longer range interactions and *trans*-interactions are possible^30^. The set of genes closest to tumour-specific IS were significantly enriched in KEGG pathways^31^ for cancer and Ras signalling, and in GO terms^32^ for leukocyte and lymphocyte differentiation (*P* **<** 0.05). There was also significant enrichment in disease gene sets (from DisGeNET^33^) related to leukaemia and lymphoma (*P* **<** 0.01), including proto-oncogenes such as *BCL2* and *MYC* (Table 1, Supplementary Table 6). Tumour-specific IS also showed two-fold enrichments in three out of five cancer gene datasets (Supplementary Table 7). Enrichments were found even though many genes in koala are not yet fully annotated. The mechanisms which may bias the genomic positions of retroviral integration are complex; however, for gammaretroviruses such as KoRV it appears likely that regions of active transcription are particularly accessible to retroviral insertion^34^. Thus, while cancer-related genes are not necessarily preferential KoRV IS, they are transcriptionally active genes playing general roles in cellular homoeostasis, growth and proliferation and are therefore likely to be accessible for gammaretroviral integration. This IS preference is likely mediated by interactions between the viral integrase and host proteins (e.g. bromodomain and extraterminal domain proteins^35^). To examine whether there was a propensity for tumour-specific IS to be located near genes with higher expression, we used published RNA-seq data from koala lymph nodes^36^ and found a small but significant shift towards higher expression levels for the set of genes closest to tumour-specific IS (*P* **=** 0.005) (Supplementary Fig. 6). Furthermore, tumour development likely elicits a selection process favouring cells containing new, somatic KoRV IS that promote their propagation and survival in a similar way to what has been observed in MLV and HIV-1 infections^37,38^, leading to selection (clonal amplification) of IS in the vicinity of such genes.

**Table 1:** Gene enrichments for sets of genes closest to tumour-specific IS.

DisGeNET	*P*	OMIM disease	*P*	KEGG pathways	*P*	GO terms	*P*
Leukemogenesis	7.99E-06	Leukaemia	0.066	Pathways in cancer	0.006	Leukocyte differentiation	0.013
Precursor cell lymphoblastic leukaemia lymphoma	5.20E-04	Lymphoma	0.260	Small cell lung cancer	0.019	Lymphocyte differentiation	0.020
Blood basophil count	7.91E-04	Protein C deficiency	0.843	Ras signalling	0.022	Lymphocyte activation	0.097
Eosinophil count result	0.0016	Rheumatoid arthritis	0.983	cGMP-PKG signalling	0.310	Regulation of response to stimulus	0.162
Eosinophil count procedure	0.0019	Gastric cancer	1.000	Kaposi sarcoma-associated herpesvirus infection	0.312	Regulation of immune system process	0.225
Leukaemia, myeloid, chronic phase	0.0019	Dementia	1.000	JAK-STAT signalling	0.314	Regulation of ossification	0.229
Carcinogenesis	0.0020	Ovarian cancer	1.000	Chronic myeloid leukaemia	0.321	Calcium-mediated signalling	0.256

We looked at and disease genes from OMIM (https://omim.org/) and DisGeNET^33^, KEGG pathways^31^ and GO ontologies^32^. *P* values are from Enrichr^76^ (calculated using Fisher’s exact test and adjusted for multiple testing).

### KoRV IS can dysregulate genes

To examine whether KoRV IS can induce gene dysregulation, we measured expression levels of genes containing tumour-specific IS in introns. We chose genes containing the highest coverage tumour-specific IS for testing (Supplementary Table 8 and Supplementary Fig 7). Using quantitative RT-PCR, we compared the expression of each gene in the tumour tissue of the koala with the IS to the same tissue type in other koalas lacking the IS. We used same tissue type to avoid among tissue variation in expression (likely to be greater than among animal variation) as a potential confounder. Gene expression could not be directly compared between healthy and tumour tissues in the same individual as distinct tissues were intentionally collected for each koala to ensure tumour-specific IS were identified (and some of the tumour types in this study were of diffuse phenotype meaning healthy matched tissue from the same organ was not available). Two additional zoo koalas were included as controls (Supplementary Table 9). These two koalas derived from a northern koala population and both died of old age (a reasonable control for the disease cohort with 40% aged animals). We found that for four of seven genes tested, expression was higher in the sample containing the tumour-specific IS compared to control tissues lacking the IS, indicating that the IS may be responsible for the change, with 4–97-fold increases (*P* **<** 0.05 for each comparison). Such changes in expression of proto-oncogenes are one mechanism by which KoRV integrations could promote tumourigenesis, malignant transformation and pro-metastasis pathways, consistent with other gammaretroviral driven expression changes^38^.

### KoRV can transduce oncogenes

**Fig. 2:**
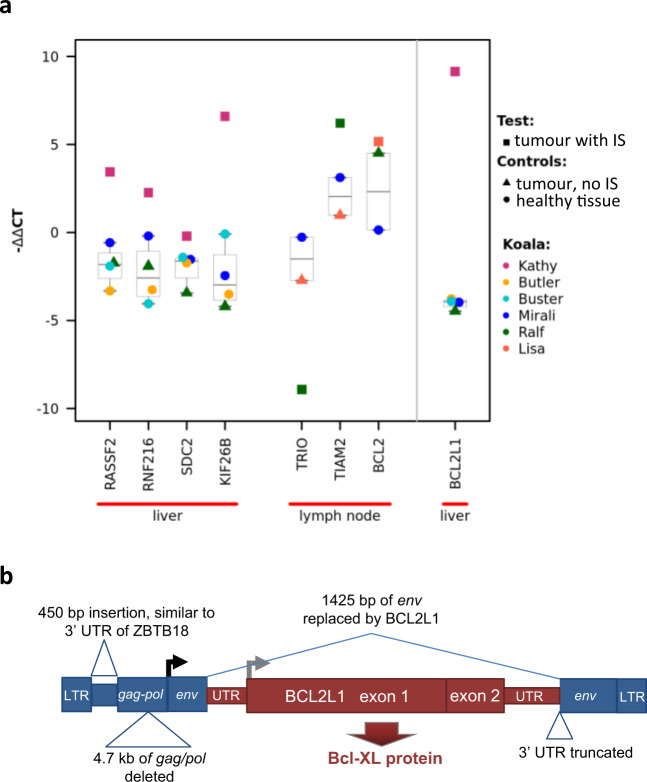
qPCR expression of genes containing tumour-specific KoRV IS and structure of KoRV containing transduced oncogene. **a** Expression of a set of genes containing tumour-specific IS. Quantitative PCR expression levels (∆∆Ct values^83^) are shown, per gene, for tumour tissue from the individual with the tumour-specific IS (square icon), and for tumour (triangles) or healthy tissues (circles) from other koalas (colour-coded). All qPCRs were performed in triplicate and the mean used for analysis. Boxplots indicate the interquartile range (IQR) with the centre line showing the median; whiskers show 1.5 × IQR. Two zoo koalas (Bilyarra and Mirali) not collected for the study of neoplasms were also used as controls, with Bilyarra used as a baseline for ∆∆Ct values (i.e. for each gene all ∆Ct values were divided by that of Bilyarra, so Bilyarra does not appear in the graph). Genes with significant changes in expression were: *RASSF2* (*P* = 0.0023), *RNF216* (*P* = 0.0096), *KIF26B* (*P* = 0.0015) and *TIAM2* (*P* = 0.0425) based on *t* tests of ∆Ct values. The expression of the transduced oncogene *BCL2L1* (*Bcl-XL*) shown on the right side of the graph was also significantly increased (*P* = 0.0004). The expression levels of *BCL2L1* were 500-fold higher in Kathy than the other koalas tested (fold change calculated as 2^−∆∆Ct^). **b** Structure of the KoRV containing transduced *BCL2L1* detected in Kathy. The two *BCL2L1* exons are joined together precisely at the splice junction and are flanked by the beginning and end of the KoRV *env* gene. The *BCL2L1* 3′ UTR joins the 5′ and 3′ ends of *env* via micro-homologies (CAG and CCTCCC, respectively). Most of *gag* and *pol* are also replaced by a sequence similar to the 3′ UTR of koala gene *ZBTB18*.

Retroviruses can acquire and transduce host genes (e.g. Rous sarcoma virus^39^, mouse gammaretroviruses^8,40^). We found evidence that this may be the case for KoRV. In one koala (Kathy), a KoRV sequence was identified in which the retroviral envelope gene was almost entirely replaced by the complete, in-frame coding sequence for the koala gene *BCL2L1* (Fig. 2b, Supplementary Fig. 8). There are two known isoforms of *BCL2L1*, a long (*Bcl-XL*) and a short (*Bcl-XS*) form, which act as an apoptotic inhibitor^41^ and an apoptotic activator, respectively^42^. The fusion gene contained the long isoform, *Bcl-XL*, overexpression of which has been found to play a pivotal role in invasion of various malignant neoplasms in other species^43^. In Kathy, expression of *BCL2L1* was found to be >500-fold greater than in other koalas (*P* = 0.00004) (Fig. 2a). Kathy suffered from anaplastic carcinoma of thyroid origin and copy number variants of *BCL2L1* have been found in this cancer type^44^. The transduced KoRV also contained part of the 3′ UTR of the koala gene *ZBTB18*.

We identified the IS associated with this transduced KoRV in Kathy and found six low coverage IS, five of which were specific to healthy tissue and one specific to tumour. This suggests multiple somatic integrations have occurred. Other somatic IS with very low read coverage may exist for the transduced KoRV, but these are difficult to detect. Overall, Kathy had the highest number of IS specific to healthy tissue (a total of 14), higher than the other koalas (which had from 0–5), indicating an abundance of somatic integrations in Kathy (Supplementary Table 3). The fact that the transduced KoRV has multiple, low coverage, tissue-specific integrations suggests that it may be exogenous. The variable parts of the LTRs and envelope protein that are used to define different KoRV strains are missing from the transduced KoRV, but the start of the LTR shows highest sequence identity to KoRV-A (98.5% across 203 bp).

We found some evidence that a similar gene transduction is also present in Elise at low titre. The *gag* gene of the transduced KoRV from Kathy also contained part of the 3′ UTR of the koala gene *ZBTB18* and this sequence was also found within the *gag* gene in reads from Elise; however, the *BCL2L1* sequence was not found in reads from Elise. Being one of the earliest collected samples, Elise had the shortest read lengths (due to DNA degradation over time) and so sequences at a greater distance from LTRs were less likely to be amplified during our iPCR procedure. Using primers specific to *ZBTB18* and *BCL2L1*, we amplified the *ZBTB18*–*BCL2L1* spanning region in Elise (using other koalas as controls), and sequencing confirmed the presence of *BCL2L1* although the junctions were not the same and this version appeared to be highly mutated compared to the version identified in Kathy (Supplementary Fig. 9). Although the data from Elise does not show that the transduced KoRV in Kathy is infectious, the low coverage, tissue-specific IS in Kathy suggest that the transduced virus may have been transmitted by infection. Further studies are required to confirm this. Although transduced retroviruses generally cannot promote infection by themselves due to loss of essential genes, they can be spread by infection using viral proteins produced by other intact proviruses within the cell^39^.

### Shared ERVs near oncogenes may predispose koalas to cancer

Of 1002 IS identified, 793 were present in both healthy and tumour samples, and represent ERVs. Each koala carried 86–117 KoRV ERVs, some of which may have the potential to contribute to gene dysregulation throughout the life of the koala. The set of genes proximate to these IS was significantly enriched in two datasets of cancer genes (Supplementary Table 7), including cancer drivers (genes such as *BRAF*, *PIK3R1*, *RELN* and *TIAM2*) suggesting that some koalas may harbour ERVs that could confer increased cancer susceptibility. We also found that the top DisGeNet enrichment for this set of genes was for body mass index (*P* **=** 0.0034), indicating that ERVs are not as strongly associated with cancer genes as tumour-specific IS.

**Fig. 3:**
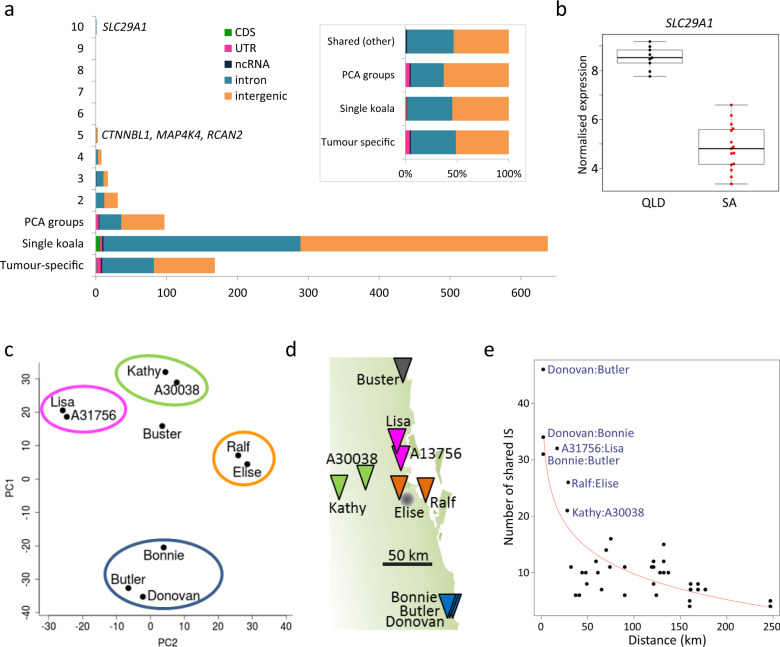
Sharing of KoRV IS. **a** Numbers of IS unique to one koala (either tumour-specific IS or IS in healthy tissue in a single koala) compared to those shared in PCA groups (shown in **c**). The single IS shared by ten koalas is located in the gene *SLC29A1*, an equilibrative nucleoside transporter. The three IS shared by five koalas were located 1 kb downstream of *MAP4K4*, in a 140 kb region between the genes *CTNNBL1* and *SRC*, and in an intron of *RCAN2*. Insert shows percentages in the categories according to genomic location. *Shared (other)* refers to all IS shared between two or more koalas that are not in PCA groups. IS shared within the PCA groups are more likely to be in exons that those shared outside the groups. **b** From published RNA-seq data^36^, the expression of *SLC29A1* in lymph nodes was found to be differentially expressed in northern koalas from Queensland (QLD) compared to koalas from South Australia (SA); this was the most significantly differentially expressed annotated gene between the two koala populations (using the Limma R package^86^ for differential expression analysis with false discovery rate used for multiple test correction: log fold change = 3.65, *P* = 1.35 × 10^−5^). Boxplots indicate the interquartile range (IQR) with the centre line showing the median; whiskers show 1.5 × IQR. **c** Sharing reflects the geographic proximity of the koalas with principle component analysis showing groups which correspond to proximity. Note: PCA analysis was carried out without prior knowledge of the geographic origin of each koala and led us to request the location data of the samples. **d** Provenance of the koalas shown on a map of coastal region bordering Queensland and New South Wales, Australia (~130 km north and south of Brisbane is shown). **e** Plot of number of shared IS versus geographical distance with pairs of koalas sharing the highest numbers of IS highlighted.

Endogenous IS are subject to gene flow and we found that 20% were present in more than one koala (Fig. 3a). All shared ERVs were found in both tissues of all koalas that shared the ERV, indicating the accuracy of ERV detection in our dataset. Only one IS was common to all of the koalas, located in an intron of the gene *SLC29A1*. This IS is also present in the koala reference genome. *SLC29A1* is a highly conserved nucleoside transporter which also plays a role in the transport of xenobiotics^45^. *SLC29A1*-null mice have a significantly lower body weight than wild-type litter mates^46^ and low expression is associated with poor prognosis in patients with hepatocellular carcinoma^47^. Using published RNA-seq data^36^, we found that this gene had significantly higher expression in northern koalas compared to southern (Fig. 3b) suggesting that the intronic IS has an effect on expression of *SLC29A1*. The presence of this ERV in all koalas examined suggests that it does not have a highly detrimental effect. No other IS was shared to the extent of *SLC29A1*; other shared IS were present in no more than five koalas, and the majority of IS were unshared.

Principal component analysis and clustering identified groups of koalas based on shared IS (Fig. 3c, Supplementary Fig. 10). These groups corresponded to the geographic provenance of the koalas (Fig. 3d), with the number of shared IS decreasing exponentially with distance (a Mantel test demonstrated a strong negative correlation: *r* **=** −0.86; *P* **=** 0.0007 (Fig. 3e)). This is consistent with the fact that koalas do not generally disperse over large distances. Within these PCA groups, koalas shared up to 43 IS (mean = 26.8) compared to nine or fewer (mean = 4.3) outside the groups (Fig. 1). Also, within the groups, we found 4.9% of shared IS were located within an exon (mainly UTRs) (Supplementary Table 10), whereas only 1.1% of IS shared by geographically separated groups were located in exons (Fig. 3a) suggesting that IS within exons are less likely to propagate widely throughout the population. There was some correspondence between the PCA groupings of the koalas and the diagnosed tumour types, despite some koalas being collected up to 10 years apart. For example, koalas Ralf and Elise both suffered from lymphoma/leukaemia and shared an IS in the 5′ UTR of *ZFAT*, a gene implicated in hematopoietic malignancies^47^. This IS was also shared by the reference genome (a female koala named Bilbo). RNA-seq data exist for Bilbo from a study of chlamydial eye disease, and using these data, we examined gene expression around the IS in *ZFAT* in Bilbo and 29 other northern koalas. We found that Bilbo and two other koalas had markedly increased expression in *ZFAT* (~5–8-fold higher) (Supplementary Fig. 11). All three koalas were collected within a ~24 km geographic radius; Ralf and Elise were the only two of our samples to also originate in this area. The increase in gene expression was found to start exactly at the position of the IS and continue throughout the *ZFAT* gene suggesting that this IS increases expression of *ZFAT*. In another PCA grouping, all three koalas (Bonnie, Butler and Donovan) developed tumours in the head and neck and shared a single exonic IS in the 3′ UTR of *LSAMP*, a gene that is a candidate tumour suppressor in human osteosarcomas^48^. We hypothesise that inherited IS in or near proto-oncogenes could predispose affected individuals to certain cancers, but a larger dataset would be needed to confirm this. Overall, ERVs that have propagated widely throughout the koala population are less likely to have a negative effect on the health of koalas, but it is possible that deleterious ERVs are inherited within groups of related koalas, particularly when the detrimental effects occur after sexual maturity.

### Hotspots of KoRV IS are located in regions containing known cancer genes

**Fig. 4:**
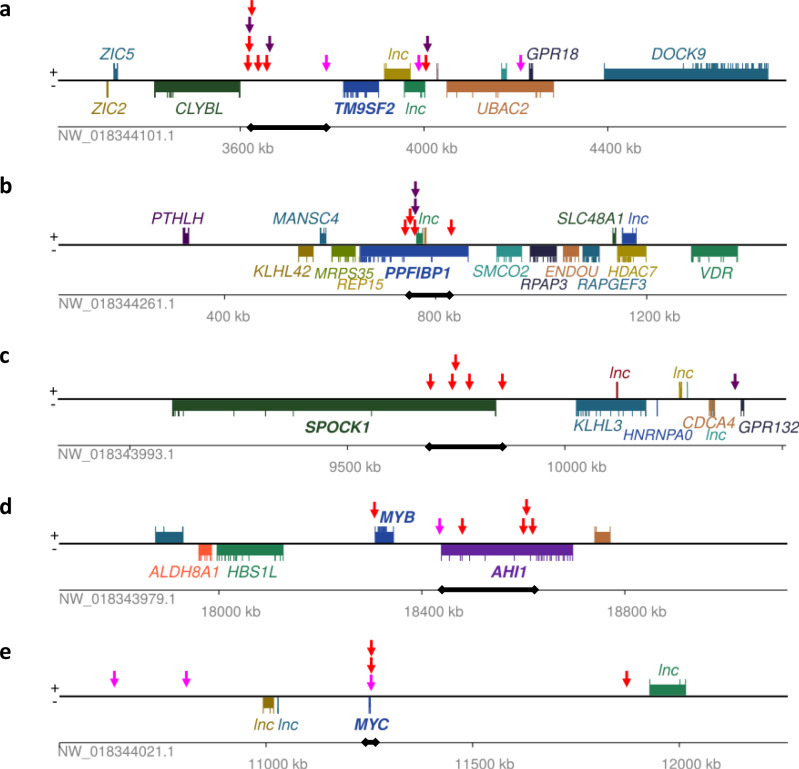
Regions with significantly high IS density (hotspots) in the koala genome. IS locations are shown with arrows. Regions longer than the identified hotspot windows are displayed to show the surrounding genes; locations of hotspots are indicated by thick back line on contig scale bar. Long non-coding RNAs are labelled “lnc” and genes without annotation are unlabelled. All regions contain multiple IS that are found in both healthy and tumour tissue (ERVs) (red arrows). Regions (**a**–**c**) contain IS that are conserved in multiple koalas (purple arrows). Regions (**a**, **d**, **e**) contain tumour-specific IS (pink arrows). All regions contain genes with associations to cancer, namely **a**, *TM9SF2*; **b**, *PPFIBP1*; **c**, *SPOCK1*; **d**, *AHI1* and *MYB*; **e**, *MYC*. There may also be positive benefits for increased expression of some genes in these regions; for example, **a** also contains a gene involved in vitamin metabolism (*CLYBL*), and the cancer genes *PPFIBP1*, *SPOCK1* and *AHI1* in regions **b**–**d**, respectively, have roles in body weight and feeding behaviour. Two other identified hotspots are not shown here because they were not in close proximity to protein coding genes: one was surrounded by non-coding RNAs and the other was at the very start of a contig (see Supplementary Table 11).

The complete set of 1002 IS detected were not distributed evenly across the genome and were found to be more often located within 10 kb of each other, at a frequency higher than expected by chance (17% of IS compared to 6.2% when positions were randomised maintaining same number of IS per scaffold, *P* < 0.0001). We used a Monte Carlo simulation method that has been previously described^38^ to determine the distribution of IS that would occur by random chance in various window sizes across the koala genome. The top seven regions of significant IS density are listed in Supplementary Table 11. These regions contain a total of 28 ERVs and 7 somatic IS. Three of these regions (Fig. 4) contained genes previously associated with cancer through insertional mutagenesis screens in mice (*AHI1*^49^, *PPFIBP1*^50^ and *TM9SF2*^51^). In such mutagenesis screens, genes involved in tumour progression are detected by the presence of multiple, independent somatic IS, with transformation most frequently induced through dysregulation of gene expression by the viral promoters^52^. *AHI1* expression is highly elevated in certain human lymphoma and leukaemia stem/progenitor cells^53^ and *TM9SF2* has been linked leukaemia^54^. *PPFIBP1* (liprin β1) interacts with metastasis-associated protein *S100A4*^55^ and has been associated with tumour progression in patients with high-grade chondrosarcomas^56^ (a malignant transformation of osteochondroma). The other hotspot regions also contained genes associated with cancer, including *SPOCK1* (Testican-1), which promotes growth, invasion and metastasis in several cancers^57,58^ including osteosarcoma^59^, and the proto-oncogenes *MYB* and *MYC*, which are frequently aberrantly expressed in leukaemias and lymphomas^60,61^. There were three independent integrations very close to the *MYC* transcription start site (clustered within 624 bp, within <1300 bp of *MYC*). Two of these were in healthy tissue and one was tumour specific. Therefore, it appears that IS with a high potential for inducing tumorigenesis can be present in all tissues. Since retroviral integrations can influence the transcriptional activity of nearby genes (on both strands), the hotspots of KoRV integration in regions containing cancer-related genes could be one factor predisposing koalas to developing tumours.

In several of the hotspots, we found that the majority of koalas contained at least one IS within the region. For example, eight koalas had at least one IS in the dense region of IS upstream of *CLYBL* (Supplementary Fig. 12). Moreover, some of the hotspots contained IS that were widely shared among koalas, and so have been subject to inheritance unlike the somatic IS detected in mouse mutagenesis screens. It is possible that a small number of IS may be selected for favourable effects in early life, while also playing a role in tumour development at a later stage, a process known as antagonistic pleiotropy^62^. For example, the region with the most significant density of IS is located between the genes *CLYBL*, which is required for vitamin B12 metabolism^63^, and *TM9SF2*, which has been associated with leukaemia^54^. Other genes in hotspots that have cancer associations might also confer positive effects. For example, *SPOCK1* and *AHI1* have associations with obesity^64,65^ and feeding behaviour^65^ and *PPFIBP1* plays an important role in the integrity of lymphatic vessels^66^, which are responsible for the uptake of dietary fat and fat-soluble vitamins. Since koalas have a nutritionally poor diet (eucalyptus), it is possible that KoRV IS, which upregulate genes involved in vitamin uptake or metabolism, and body weight or feeding behaviour could be beneficial even if there may be detrimental effects later in life.

## Discussion

**Fig. 5:**
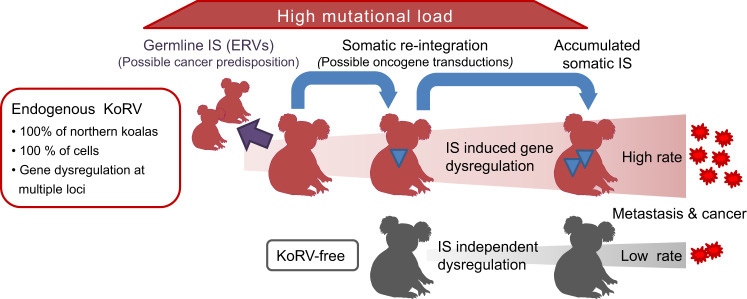
Overview of KoRV transformation processes in koalas. All northern koalas carry endogenous KoRVs (red koala images) which are subject to Mendelian inheritance (purple arrow). Having multiple potentially active ERVs in every cell, with reintegration (blue arrows) leading to additional IS in somatic cells (blue triangles), would confer a tremendous mutational load on the population. Both inherited and new somatic IS can potentially dysregulate oncogenes, leading to an increased risk of transformation, with additional new integrations occurring in the transformed population of cells promoting cancer and metastasis. The potential for KoRV to transduce oncogenes also increases cancer risk. Koalas without endogenous KoRV (grey koala images) will only develop cancers (at a much lower rate) through other mechanisms.

We provide insights into the ongoing retroviral invasion of the koala genome, focusing on the potential of KoRV to underlie the high cancer rates in the northern koala population. Using a method to accurately and comprehensively detect KoRV IS, we examined both tumour and healthy tissues from ten koalas, allowing us to detect KoRV ERVs and somatic integrations in tumour tissues. We find that northern koalas carry a high mutational burden from a rapid expansion and accumulation of endogenous KoRV integrations in the population. All koalas in the northern population likely carry similar numbers of KoRV-A proviruses^67^ with some proviral IS possibly conferring a higher cancer risk than others. It is also clear that this germline mutational load is compounded in individual koalas by substantial accumulation of somatic integrations. We find evidence that KoRV can induce gene dysregulation of proximate genes, both as an ERV and following somatic integrations. The inherited KoRV ERVs present in all tissues provide a pool of proviruses in every cell, from which new somatic integrations or gene transductions could potentially occur, with some somatic reintegration events potentially affecting oncogenes. KoRV-mediated dysregulation of oncogenes may cause cells to clonally expand resulting in our observed enrichment of tumour-specific integrations near oncogenes. In addition, we find that KoRV is able to transduce host oncogenes, potentially resulting in a transforming virus that could be transmissible, which would be of concern for koala conservation efforts. Although other factors may also contribute to cancer in koalas, the mutational burden from endogenous KoRV likely increases the frequency of tumorigenesis and may potentially shorten the time for cancer to develop (Fig. 5).

Koalas and KoRV represent one of the youngest germline invasion processes observed in mammals (<50,000 years) demonstrating the effects on the host at the earliest stages of the process, when the exogenous retroviruses and ERVs are biologically most similar. In most other mammalian species, ERVs are derived from germline invasions that occurred millions of years ago, so that host genomes now only contain ERVs that are fixed and inactive. A notable exception to this are MLVs in wild and laboratory mouse strains which remain capable of expression and replication even after ~1.5 million years within the host genome^11^. However, the variation in MLV insertion number, location and activity in mice is less than that of KoRV in koalas, probably reflecting, in part, the increased time frame of association of MLVs with their host. In species with much older ERVs (e.g. human ERVs^68^), the mutational load of these ERVs appears to be very low and few are linked to cancer in individuals.

In koalas, which have very high cancer rates for mammals, the KoRV-A induced mutational load should over time be purged, in part by their gradual degradation through mutation or recombination^9^, with degraded ERVs having a less detrimental impact on the health of the host. It is these degraded ERVs that are likely to persist and become fixed in the genome. However, koalas are at such an early stage in the endogenisation process^10^ that the tipping point at which the exogenous retroviral counterpart becomes extinct, degraded retroviruses cease to genomically proliferate, and subsequent genomic fixation occurs may not be reached for hundreds of thousands of years. Surviving retroviral endogenisation also involves the host genome accommodating the endogenous retroviral content or mitigating its effects, contributing to the evolution of host viral suppression mechanisms, for example, piRNAs^69^ or APOBEC3G^70^.

Overall, the newly endogenised retroviral content substantially affects the host genome in multiple ways, not only with strong selection pressure likely arising due to negative health effects but also with ERVs contributing new genomic sequence such as promoters, and even providing some beneficial effects^71^. This renders the evolutionary dynamics between virus and host complex. The profound evolutionary consequences of germline invasions have shaped vertebrate genomes and have been repeatedly experienced during vertebrate evolution, including the lineage leading to modern humans. However, the immediate health effects on a species, including increased cancer rates, must first be endured and overcome.

## Methods

### Sample collection

Koalas are a vulnerable species and northern koalas in particular suffer from multiple widespread diseases (e.g. KoRV related wasting, endemic chlamydia and cancer) and are also prone to injuries (dog attacks and road accidents) and so are regularly admitted to dedicated wildlife facilities for treatment and rehabilitation. The koalas included in this study were admitted to dedicated wildlife treatment and rehabilitation centres after being rescued from the wild for overt illness (e.g. large tumours, wasting). All treatment facilitates perform thorough and consistent veterinary examination and diagnostic procedures on all admissions. Note: koalas are given unique identification numbers at admission but are also commonly named for ease of identification by the veterinary team. After undergoing thorough diagnostic procedures under general anaesthetic performed by highly experienced wildlife veterinarians, koalas were diagnosed with advanced/untreatable neoplasia. Due to the serious and untreatable nature of their condition, koalas were humanely euthanised by the veterinary team via intravenous injection of pentobarbitone (0.5 ml/kg). After confirmation of death (listening for cessation of heartbeat), the koalas were promptly necropsied and tissue samples were collected during routine post mortem procedures by the onsite wildlife veterinarian. Individual tissues of interest were collected for histopathology (5–8 mm in size put into 10% buffered formalin) and immediate freezing (−80 °C) for later assessment.

Sample collections were conducted in accordance with the University of Queensland Animal Ethics Committee (approval no. MICRO/PARA/612/08/ARC).

### DNA extraction and quantification

Overall, 30 mg of paired tumour and healthy tissue from each individual koala (Supplementary Table 1) was extracted using the Qiagen DNeasy^®^ Blood and Tissue Kit (Qiagen, Hilden, Germany) according to the manufacturer’s protocol with the following modifications: the tissue was lysed overnight for 15 h at 56 °C and hair was lysed for 48 h at 56 °C. The DNA concentration and quality was then determined using an Agilent Tapestation (Agilent Technologies USA) with Genomic ScreenTapes and reagents.

### Shearing of DNA and circularisation of fragmented DNA

The genomic DNA for paired tumour and healthy tissues (Supplementary Table 1) was sheared to between 3 and 4 kb using the Covaris™ M220 with miniTUBE Blue. All extracted samples were fragmented using the following customised settings: intensity 0.1, 20% duty factor, 1000 cycles per burst, ten cycles at 20 s per cycle, temperature 20 °C. Where possible, 3 µg total DNA was fragmented and if not available, 1 µg total DNA was used in a final volume of 100 µl. To find the optimal ligation conditions for subsequent iPCR, we performed a series of ligations using a gradient of (total) input blunt ended DNA. This consisted of three gradient points (20, 40 and 60 ng of total DNA) representing concentrations of 0.2, 0.4 and 0.6 ng/μl run in triplicate 50 µl ligation reactions. Each ligation reaction was set up using a T4 DNA Ligase kit (5 U/µl) (Thermo Scientific) using 5 µl of T4 DNA ligase buffer (10X), 5 µl of 50% PEG 4000 solution, 2.5 µl of T4 DNA ligase and molecular biology grade water (Thermo Scientific). To reduce any sampling biases, each sample was diluted down to a concentration of 10 ng/μl (as per TapeStation readings) and the correct volume of DNA for each gradient point added accordingly. A circularisation (non-template) water blank was also run in triplicate for all samples. All circularisation reactions (samples and controls) were performed in triplicate and then pooled.

### Inverse PCR

A total of 10 ng from each of the circularised gradient points was used as template for quadruplicate iPCR reactions. iPCR reactions were then performed on all paired healthy and tumour tissue samples (fragmented and circularised) using the following LTR primers: forward 5′-ATT TGC ATC CGG AGT TGT GT-3′ and reverse 5′-AGG GGC ACC CTA GAA ACT GT-3′. All primers are listed in Supplementary Table 12. Each primer contained a unique 6 bp barcode with additional three sacrificial nucleotide bases (to prevent loss of the barcode in case of limited DNAse activity) at the 5′ end to enable identification in case samples were later pooled for sequencing (although most samples were run in separate cells on a PacBio RSII machine). All samples and controls were amplified using the MyFi Mix (Bioline GmbH, Luckenwalde, Germany) using 50 µl of MyFi buffer (2X) and a final 0.4 µM concentration of each indexed primer in a 100 µl volume. Thermal cycling conditions were as follows: 95 °C for 1 min 30 s; followed by 30 cycles at 95 °C for 20 s, 57 °C for 30 s and 72 °C for 3 min. The iPCR products from the quadruplicate reactions were pooled for each gradient point and purified using the QIAquick PCR Purification Kit (Qiagen, Hilden, Germany). Concentrations and DNA profiles were measured on the TapeStation and the optimal circularization product from all sample-amplified gradients (20, 40 or 60 ng) was selected by considering three criteria: (i) the DNA amount per microliter of iPCR product in the 2–7 kb range, (ii) the average length distribution between 600 bp and 7 kb range and (iii) the percentage of DNA within the 2–7 kb range. Only the optimal circularisation gradient point for each sample was used for PacBio sequencing.

### PacBio sequencing

The purified iPCR products from the healthy and tumour tissues were processed by the Max Delbrück Center, Berlin, for PacBio library construction and sequencing. Samples were individually purified using AMPure XP beads (Beckman Coulter), at a concentration of 0.9X. Libraries were then created for each sample using the PacBio (Pacific Biosciences, Menlo Park, CA) 5 kb template preparation protocol and the SMRTbell™ Template Prep Kit 1.0 following the manufacturer’s guidelines. The length and concentration of the libraries were then determined with the 2100 Agilent Bioanalyzer using the 1200 DNA chemistry (Agilent Technologies). Sequencing on the PacBio RSII platform was performed using the MagBead Standard protocol, C4 chemistry and P6 polymerase on a single v3 Single-Molecule Real-Time (SMRT) cell with 1 × 180 min movie for each sample. Every sample was run across two Single-Molecule Real-Time (SMRT) cells to increase the coverage and an extra sequencing run was done for Elise tumour tissue (multiplexed with other samples) to further increase coverage. The reads from the insert sequence were processed within the SMRT^®^ Portal browser (minimum full pass = 1; and a minimum predicted accuracy of 90).

### Detecting IS from iPCR reads

Parts of the PacBio reads mapping to the KoRV-A or KoRV-B genomes (GenBank: AB72100.1 and KC779547.1, respectively) were identified using BLAST^72^ and removed so that only the koala genomic sequence surrounding the insertion sites remained. The published koala genome (*P. cinereus* release phaCin_unsw_v4.1, RefSeq assembly GCF_002099425.1) was also masked for KoRV sequences. The trimmed reads were aligned to the masked koala genome using BWA^73^ with the following parameters: BWA MEM –x pacbio –c 2500. The results were filtered using Samtools^74^ to remove reads that failed platform/vendor quality checks or were PCR duplicates. Matches longer than 50 bp with >90% identity to the koala genome were retained. Retroviral IS have a distinct coverage pattern from iPCR sequencing (Supplementary Fig. 2b). We used a stepwise approach whereby: (i) high coverage IS with at least ten reads on each side were identified (≥20× coverage at overlapping read starts/ends within a 10 bp window) and corresponding reads removed from the dataset; (ii) low coverage insertion sites (at least two reads on each side with at least five reads in total) were identified and corresponding reads removed from the dataset and (iii) one-sided IS (where IS had reads mapping only to either the upstream or downstream sequence) were identified from the remaining reads. The one-sided insertion sites were checked to see if there were both sides (a start and end) within 10 kb, indicating that the published koala genome also contained a KoRV at these positions or that there was an indel in the koala genome between these positions. The level of sequencing allowed us to reach almost full saturation of germline insertion sites in both tumour and healthy tissues (Supplementary Fig. 3).

### Comparing tumour and healthy samples

For each koala, the coordinates of the IS from tumour tissue were compared to those from healthy tissue (using bedtools merge from the Bedtools suite^75^) to find IS unique to one tissue and IS common to both tissues. IS which overlapped or were within 10 bp of each (since the boundaries of the IS can vary slightly due to alignments) were annotated as the same IS. Scatter plots of insertion site coverage comparing tumour to healthy tissue in each koala were generated.

### PCR to confirm tumour-specific IS

Seven of the highest coverage IS specific to tumour tissues were chosen for PCR confirmation. All primers are listed in Supplementary Table 12. The large number and generally high frequency of flanking sequences with large homopolymer stretches or skewed GC content precluded designing primers for many tumour-specific integrations. Primers were designed against the genomic region surrounding the IS (within 200 bp 5′ or 3′ of the IS). For each IS, one koala genome primer and one LTR primer was used in order to cross the IS (Supplementary Fig. 4a). The target sequence was amplified in the following reaction: 12.5 µl of MyTaq™ HS Mix (Bioline, USA), 3.5 µl of PCR grade water and 1.5 µl (10 mM) of IS specific primer and 1.5 µl (10 mM) or either forward (5′-ATT TGC ATC CGG AGT TGT GT-3′) or reverse LTR (5′- AGG GGC ACC CTA GAA ACT GT-3′) primers. The following thermocycling conditions were used: (i) 95 °C for 5 min; (ii) 35 cycles, with 1 cycle consisting of 95 °C for 20 s, 60 °C for 20 s and 72 °C for 1 min and (iii) 72 °C for 2 min. The resulting amplicons were Sanger sequenced at LGC Genomics (Germany) by primer walking and the sequences checked using BLAST^72^ to compare them to the expected IS and adjacent KoRV LTRs.

### Detection of KoRV variants

The *env* gene sequences from the KoRV variants described in Chappell et al.^14^ (108 sequences from KoRV variants A, B, D, F, G, H, I) were downloaded from NCBI and the most highly variable section from the published sequence alignment was extracted (base 251 onwards). We used BLAST^72^ with an *e*-value cut-off of 0.001 and minimum match length of 90 bp to compare the variable region to all koala reads.

### Annotation of insertion site locations and gene enrichment analyses

Bedtools closest from the Bedtools suite^75^ was used to annotate the location of each IS with reference to the koala transcriptome annotation (version 4.1). We took the closest gene to each IS, regardless of whether sense or antisense strand. The gene set was split into categories according to the category of the closest IS, namely: tumour unique; healthy unique (unique to a single koala) and shared. The position of IS relative to genes (distance 5′ or 3′, in exon, intron or UTR) was annotated.

We used Enrichr^76,77^ to detect any gene enrichments in KEGG pathways^31^ and OMIM and DisGeNET^33^ disease gene sets (using Fisher’s exact test). Since these enrichment analyses were carried out on human gene sets and not all human genes are annotated in koala, we repeated the analysis on DisGeNET to check for any bias as follows: the full DisGeNET gene sets were downloaded and only genes common to human and koala were selected. The tmod package in R was used to perform a hypergeometric test using the koala IS genes as the target set and the modified DisGeNET sets as the background. The disease enrichments and *P* values were compared to those from Enrichr and were found to be highly similar with the same top enrichments. GO Ontology enrichments were carried out using GOrilla^78^ using only genes common to human and koala in both target and background sets. We downloaded five databases of cancer genes: Bushman Lab Cancer Gene List (http://www.bushmanlab.org/links/genelists, v4 May 2018), harmonizome lymphoma^79^, Intogen^80^, DriverDB^81^ and The Cancer Gene Atlas (TCGA)^82^. We used these to detect cancer gene enrichments in the sets of genes closest to healthy tissue IS and tumour-specific IS. For genes from TCGA (variants across 10,000 patients in 33 cancer types), only genes with variants found in > 500 individuals were used.

### RNA Extraction and cDNA synthesis for quantitative PCR (qPCR)

Overall, 30 mg of paired tumour and healthy tissue from each individual koala was extracted using Qiagen RNeasy^®^ Mini Kit (Qiagen, Hilden, Germany) with the RNase-Free DNase Set. The following modifications were made to the manufacturer’s protocol: tissues were lysed using 600 µl RTL buffer and the Precellys 24 Tissue Homogenizer. cDNA was then generated from the extracted rRNA using the Invitrogen SuperScript^®^ IV Kit (Thermo Fisher Scientific, USA) following the manufacturer’s instructions; two reactions per sample were performed. Second strand synthesis was then carried out by adding 1 µl of Klenow DNA polymerase I (Thermo Fisher Scientific, USA) to 21 µl of cDNA and incubated at 37 °C for 60 min followed by 75 °C for 20 min. The cDNA was the quantified using the Agilent Tapestation (Agilent Technologies, USA) using D1000 ScreenTapes and reagents.

### Selecting genes for qPCR

From the six koalas that had available tissue for expression analysis, we chose the three koalas with the largest numbers of tumour-specific insertion sites for qPCR analysis. The list of IS was ordered by the read coverage (as very low coverage may indicate that the insertion site is not in many cells in the tissue). Insertion sites were excluded if only one side of the insertion site was covered by reads. We selected genes where the insertion site was in an intron (as we make the assumption that this gene will be most affected by any promoter effects). We excluded genes which had putative annotations or were of unknown function.

To choose regions for primer design, we used the koala transcriptome annotation to select consecutive exons that were present in all predicted transcript variants of each gene to avoid alternative splicing events. In case of any errors in the koala genome annotation, we used Blastx to compare these exons to the SWISSPROT/TREMBL database to select exons which are conserved in other species at the protein level. Forward and reverse primers were placed in consecutive exons.

### Gene expression using qPCR

To assess gene expression levels, SYBR green based comparative CT (ΔΔCT) and qPCR were employed. Koalas selected were those that had tissue samples from liver and/or lymph node so that expression levels could be measured in the same tissue between koalas. Two additional koalas from outside this study (Bilyarra and Mirali) were also included as controls. Expression levels of seven genes in healthy and tumour tissues were assessed. The samples tested in each case were: (i) tumour tissue from koala with IS in gene of interest; (ii) tumour tissue from koalas without IS in gene of interest and (iii) healthy tissue. Primer concentrations were optimised according to the manufacturer’s protocol. All primers are listed in Supplementary Table 12. Overall, 100 ng of cDNA from both tumour and healthy tissues were run in triplicate reactions for each primer set in addition to negative controls and housekeeping gene controls. The target gene was amplified using the following reaction: 0.5 µM of the forward and reverse primer, 12.5 µl SYBR Green JumpStart Taq ReadyMix (Sigma-Aldrich, USA), 0.25 µl reference dye (ROX) and 10 µl of template cDNA. The PCR was carried out in 96-well plates using the Stratagene MX3000P System (Agilent Technologies, USA) and StepOnePlus instruments under the following cycling conditions: 94 °C for 2 min, followed by 40 cycles, with 1 cycle consisting of 94 °C for 15 s and 60 °C for 1 min. GAPDH was run as a housekeeping gene. For all koalas, ∆CT were calculated by subtracting the CT value of GAPDH from the test gene CT value. Gene expression values were normalised (∆∆CT) by subtracting the ∆CT of Bilyarra’s healthy tissues. The fold difference was then taken as 2^−∆∆CT^^83^.

### Analysis of shared IS

PCA was performed on a matrix of IS versus koalas for all IS in healthy tissues using the R package logisticPCA for dimensionality reduction of binary data. Clustering was performed using hclust in R with a binary distance matrix. Geographic distances between koalas were calculated as the shortest straight line between locations. A Mantel test of distance versus number of shared IS was performed in R using the ade4 library.

### Expression of *SLC29A1* and *ZFAT*

Data were downloaded from the European Read Archive (Project numbers PRJEB21505 and PRJEB26467, respectively). Reads were trimmed using BBduk from BBTools (https://sourceforge.net/projects/bbmap/) and aligned to the koala reference genome (RefSeq assembly GCF_002099425.1) using STAR^84^. Reads were counted using htseq-count^85^ and differential expression was analysed using the R package Limma^86^, with sex and disease status included as factors in the model. As a further check for *SLC29A1*, the expression was normalised using the expression of the housekeeping gene *GAPDH* in the samples but the difference in expression between northern and southern koalas remained.

### Retrovirus transduction discovery

The transduced *BCL2L1* was discovered because reads mapping to this gene had an unusual coverage pattern where the reads mapped precisely to the exons, ending at the exon boundaries. From the full-length reads, the fusion of *BCL2L1* within the KoRV *env* gene was apparent and, additionally, a fragment of the 3′ UTR of the gene *ZBTB18* was also detected in the *gag* gene. All koala samples were subsequently searched for this fusion using BLAST and six koalas (Elise, Lisa, Bonnie, Ralf, A30038 and A31756) were checked using PCR. All primers are listed in Supplementary Table 12. The band from PCR of Elise was Sanger sequenced.

All samples were also scanned for any other reads which mapped precisely to exons, but none were found. Integrations sites for the transduced virus were identified by selecting the reads containing to the *BCL2L1* fusion and removing any parts matching the *BCL2L1* fusion sequence. BLAST^72^ was used to find where the remaining parts of the reads mapped in the koala genome.

### Distribution of IS in koala genome

To assess if IS were distributed in a random fashion, we performed 1000 randomizations of IS coordinates using bedtools shuffle^75^ and calculated the numbers of IS located within 10 kb. We then performed a chi-squared test for 2 × 2 contingency tables comparing real IS to random IS on number of single IS in 10 kb compared with number of two or more IS within 10 kb.

### Finding statistically significant hotspots of IS

We implemented a Monte Carlo simulation following a method used to find common insertion sites in a retroviral tagging study^38^. We used bedtools shuffle^75^ to generate 10,000 randomisations of the IS across the koala genome. For windows of varying sizes (2, 10, 30, 50, 100, 200, 300 and 400 kb), we counted the number windows containing a given number of IS (2–10). Also, since a random distribution of IS in the genome follows a Poisson distribution, we calculated the Poisson probability for each window size containing two or more IS and corrected for number of windows tested.

### Reporting summary

Further information on research design is available in the Nature Research Reporting Summary linked to this article.

## Supplementary information

Supplementary Information

Descriptions of Additional Supplementary Files

Supplementary Data 1

Reporting Summary

## Data Availability

Sequence data that support the findings of this study are deposited in the NCBI Short Read Archive under BioProject PRJNA587467. The remaining data are available within the article, Supplementary Information or available from the authors upon request.
