# Supplementary material for: Retroviral integrations contribute to elevated host cancer rates during germline invasion
Source: Nat Commun. 2021 Feb 26;12:1316. doi: 10.1038/s41467-021-21612-7 (PMC7910482; doi:10.1038/s41467-021-21612-7)
Supplement: Supplementary file 1 — Supplementary Information [file 41467_2021_21612_MOESM1_ESM.pdf]

Supplementary Information

Retroviral integrations contribute to elevated host cancer rates during germline invasion

Gayle K. McEwen, David E. Alquezar-Planas, Anisha Dayaram, Amber Gillett, Rachael Tarlinton, Nigel Mongan, Keith J. Chappell, Joerg Henning, Milton Tan, Peter Timms, Paul R.Young, Alfred L. Roca, Alex D. Greenwood*

*Correspondence to: greenwood@izw-berlin.de

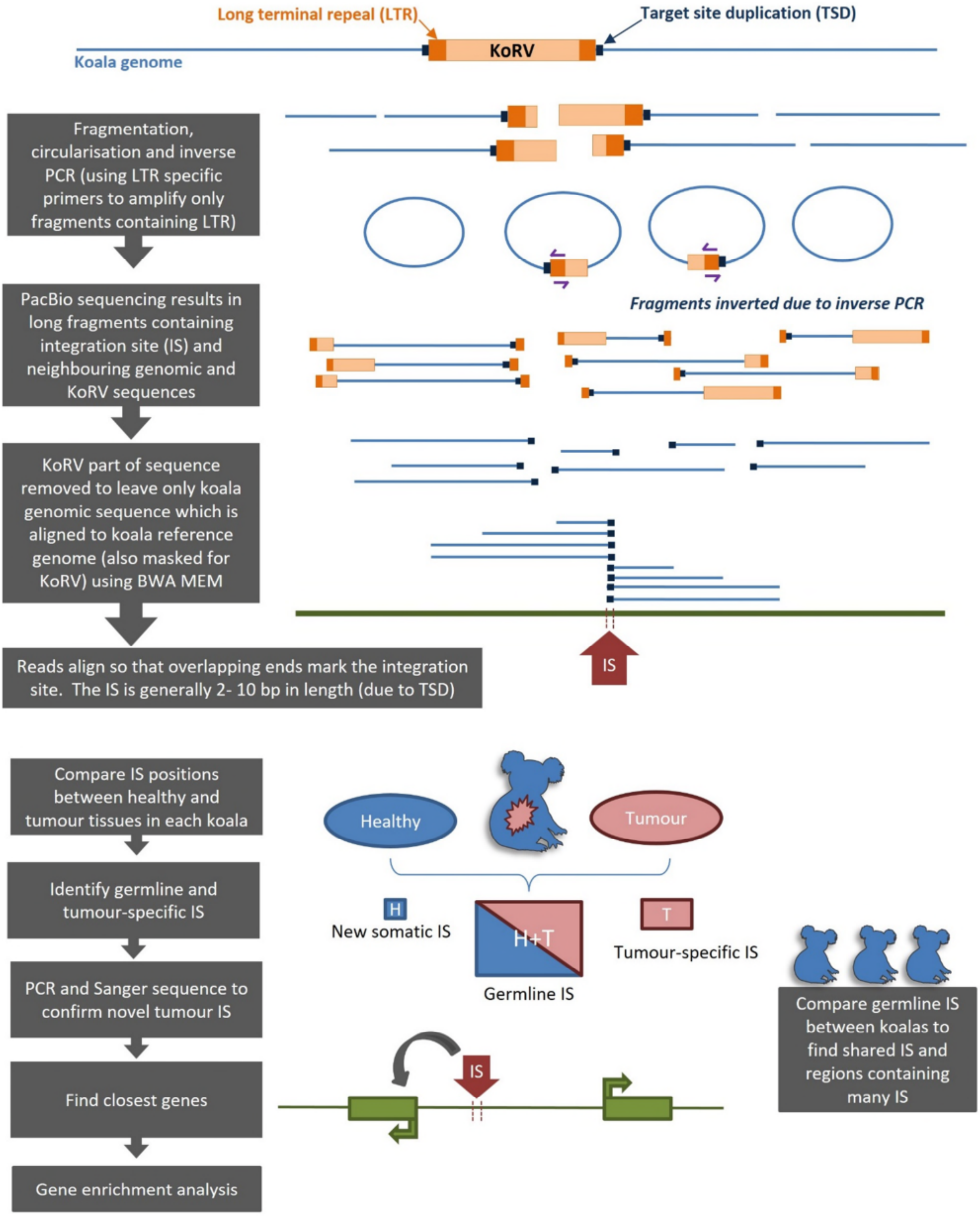

Long terminal repeal (LTR)
Target site duplication (TSD)
KoRV
Koala genome
Fragmentation, circularisation and inverse PCR (using LTR specific primers to amplify only fragments containing LTR)
Fragments inverted due to inverse PCR
PacBio sequencing results in long fragments containing integration site (IS) and neighbouring genomic and KoRV sequences
KoRV part of sequence removed to leave only koala genomic sequence which is aligned to koala reference genome (also masked for KoRV) using BWA MEM
Reads align so that overlapping ends mark the integration site. The IS is generally 2- 10 bp in length (due to TSD)
IS
Compare IS positions between healthy and tumour tissues in each koala
Healthy
Tumour
Identify germline and tumour-specific IS
H
New somatic IS
H+T
Germline IS
T
Tumour-specific IS
PCR and Sanger sequence to confirm novel tumour IS
Compare germline IS between koalas to find shared IS and regions containing many IS
Find closest genes
IS
Gene enrichment analysis

Supplementary Fig. 1| Workflow for identifying genomic locations of integration sites and subsequent analysis. Our method[1] differs from previously published methods[2] as follows: (i) fragmentation is random (sonication) rather than using restriction enzymes; (ii) no linker sequences are required for iPCR; (iii) PacBio long read sequencing (generating fragments of several kilobases in length) avoids spurious alignments to incorrect regions of the genome, and (iv) since reads traverse the ends of the integrated provirus there is no requirement to infer integration sites as would be the case for short paired-end reads (IS are easily identified because of the clear pattern of read coverage at the integration site as shown in Supplementary Figure 2).

To further limit coverage biases and false positives and negatives: (i) all samples were prepared in triplicate (sonication and iPCR) and then pooled for sequencing; (ii) the koala genome and the iPCR fragments were masked for KoRV and recKoRV[3] before alignment to reduce the chance of false hits due to these sequences.

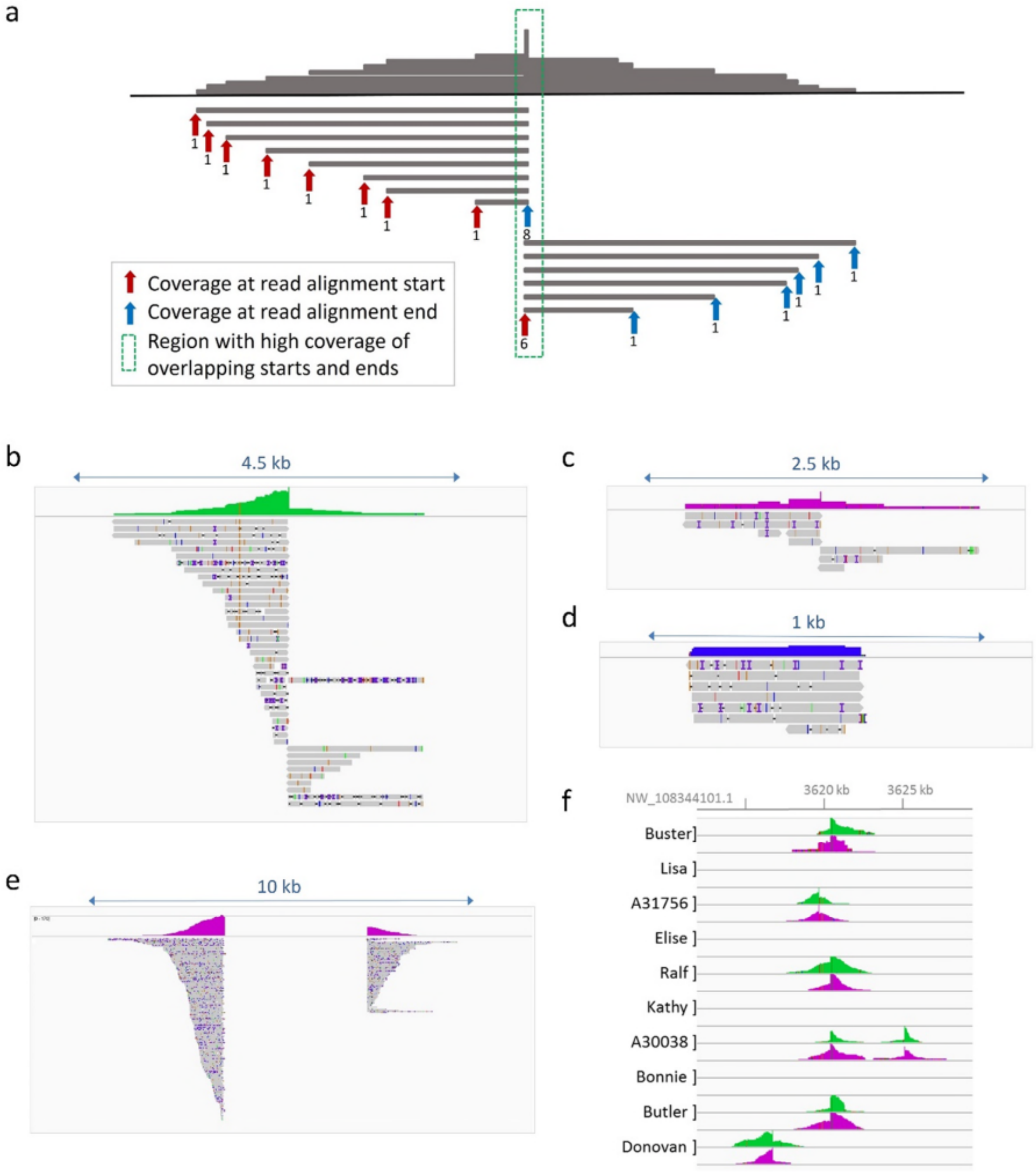

Supplementary Fig. 2| Pattern of sequence alignment around KoRV integration sites from reads generated by inverse PCR. a, IS show a distinct pattern of read coverage using our method[1]. The coverage gradually increases towards the integration site with the highest coverage at the IS. At the integration site there is usually a small target site duplication (TSD) of 2 to 10 bp where reads may overlap, causing a small spike in coverage; real IS almost always contain an obvious TSD which helps to distinguish them from spurious alignments (although spurious

alignments are not common due to length of reads). Only coverage at reads ends was used to identify IS as shown by red and blue arrows for 5' and 3' ends respectively. **b**, Most IS had coverage of > 20X at the target site duplication and an example is given. **c**, Some IS had low coverage (possibly only present in some cells of the sample), but if they contained the correct pattern of reads and insertion site duplication they were also identified as IS. Low coverage insertion sites were checked in more detail to make sure that they were not sequencing or alignment artefacts. **c** is an example of a low coverage IS and **d** is a low coverage region not identified as an IS. **e**, Although most IS have reads mapping to both sides, some insertion sites were "one-sided" but still had a similar pattern with read coverage gradually increasing towards it IS (on one side only) and stopping abruptly at the IS. We checked if there is another "one-sided" IS within 10 kb, as was found in example **e**. It is assumed that this is a single IS and there is an indel in the sample compared to the reference genome (or reference genome also has an IS here). **f** is an example a region where there is an IS (ERV) that is shared between three koalas together with 3 other IS that are specific to single koalas. Figures produced using the Integrative Genomics Viewer[4].

For all samples, we compared the read coverage from aligned reads at all positions to the locations of identified IS to highlight any regions of high coverage where no IS had been identified. Here we found 9 IS that could be added to the identified IS. Therefore, ~ 1.6 % of sequenced ERVs were missed using our pipeline, but were subsequently added. We also found two high coverage regions in the gene *BCL2L1* that did not match the expected read pattern from IS; this turned out to be a gene transduction event.

Note that the empty site for heterozygous individuals cannot be detected as the inverse PCR method employed uses primers in the retroviral LTR and therefore we cannot tell if IS are homozygous or heterozygous without further targeted sequencing.

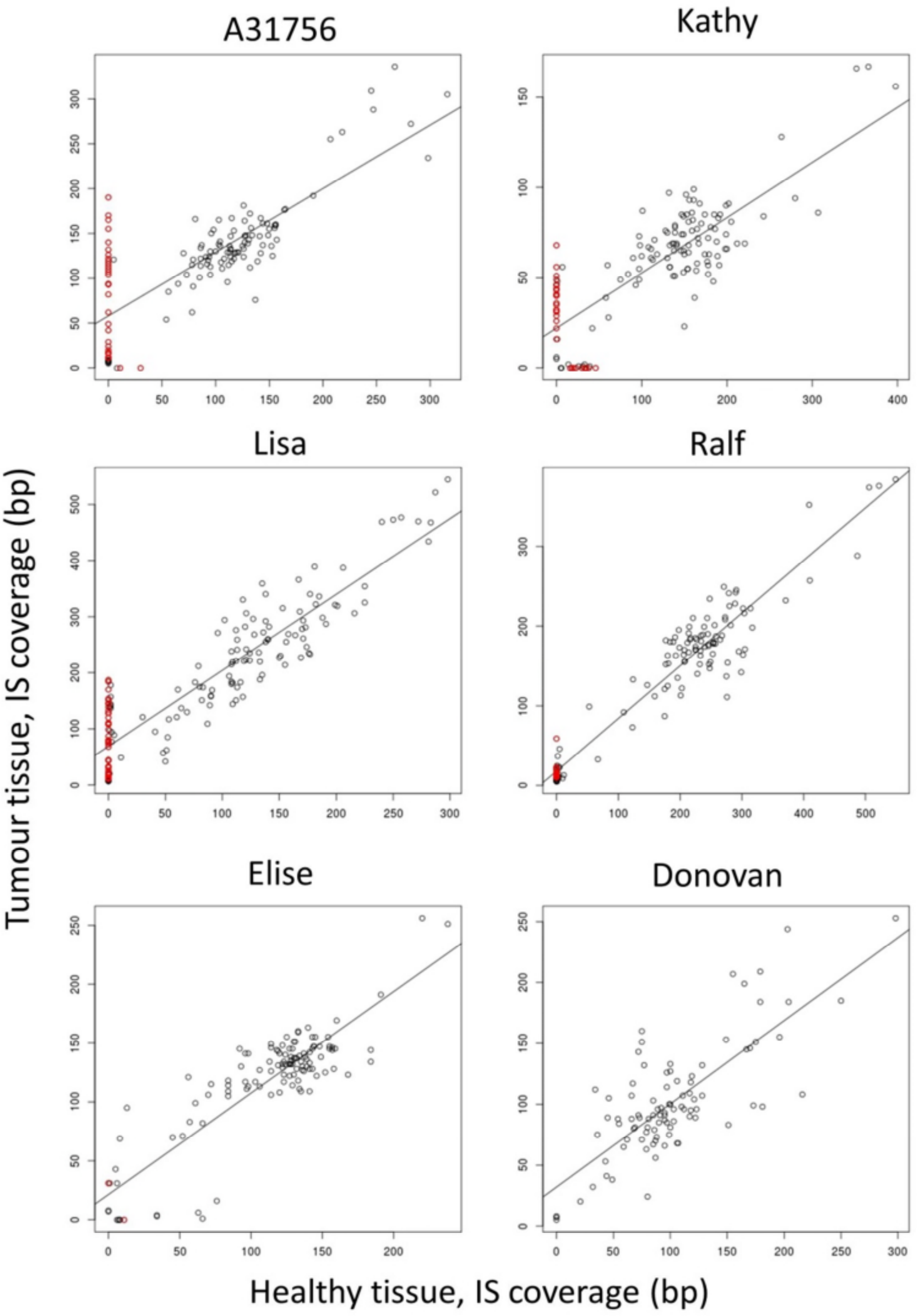

Supplementary Fig. 3| Comparison of coverage of integration sites between healthy and tumour tissue. The four koalas with the highest numbers of tumour-specific IS are shown along with two containing low numbers of tumour-specific IS for comparison. Each point represents a single IS and coverage is measured at the target site duplication. In general the coverage values are well-correlated showing that detection of IS is almost saturated and differences in overall sequencing depth are not the reason for detection of tumour-unique IS. The fact that the majority of IS were present in both healthy and tumour tissues in single koalas also indicates few false

positives or negatives in our data since these IS were detected independently in two tissues. Overall, we found almost 5 times as many tumour-specific IS than healthy-specific IS, and this was as expected under the assumption that new somatic IS are more likely to occur in tumour tissue than healthy tissue. IS with zero coverage in one tissue are indicated in red.

a

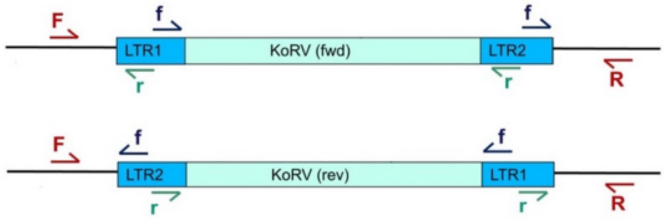

b

Num.	Koala	Sample
1	-	H_2O
2	Lisa	Healthy
**3**	**Lisa**	**Tumour**
4	Kathy	Healthy
**5**	**Kathy**	**Tumour**

c

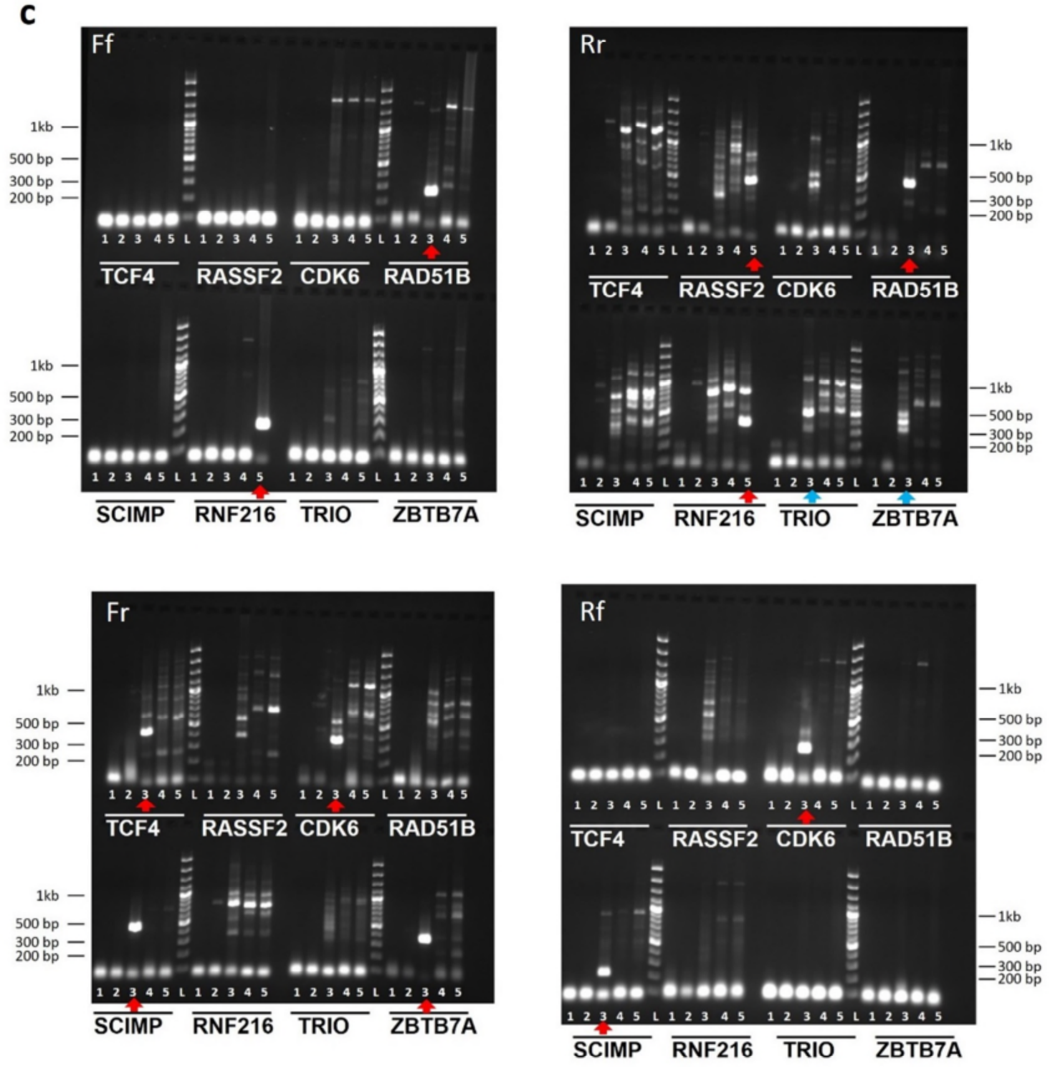

d

		Tumour specific	Correct koala	End	Direction of IS
Lisa T	TCF4	Yes	Yes	5’	fwd
	CDK6	Yes	Yes	Both	fwd
	RAD51B	Yes	Yes	Both	rev
	SCIMP	Yes	Yes	Both	fwd
	ZBTB7A	Yes	Yes	5'	fwd
Kathy T	RASSF2	Yes	Yes	5’	rev
	RNF216	Yes	Yes	3’	rev
Ralf T	TRIO	Negative control	NA	NA	NA

Supplementary Fig. 4| PCR confirmation of tumour-specific IS. a, Possible combinations of primers that would cross the integration site. These depend on the orientation of KoRV relative to the genome. Lowercase f/r = forward/reverse primer in KoRV LTR; uppercase F/R = Forward/Reverse primer in koala genomic sequence upstream or downstream of IS (designed specifically for each IS tested); We expect pairs of bands *only* in the following combinations: Fr and Rf (= KoRV in forward orientation) or Ff and Rr (= KoRV in reverse orientation). Expected band sizes are (i) for Ff and Rf: 83 bp plus distance from insertion site to KoRV primer, and (ii) for Fr and Rr: 240 bp plus distance from insertion site to primer for Fr and Rr. **b,** Samples: tumour and healthy tissues from Kathy and Lisa were used. **c,** Gel photos for each combination of primers (Ff, Rr, Fr, Rf) for each gene in each sample tested. Numbers 1 to 5 correspond to samples in **b** and L = 100 bp DNA ladder. Arrows show products that were Sanger sequenced: red = expected bands that gave correct sequence, blue = spurious bands that gave incorrect sequence. **d**, Summary of results indicating the tumour-specificity of each IS tested. Since the PCRs produced the expected results for every gene and the expected bands gave the correct sequence, this experiment was only performed once.

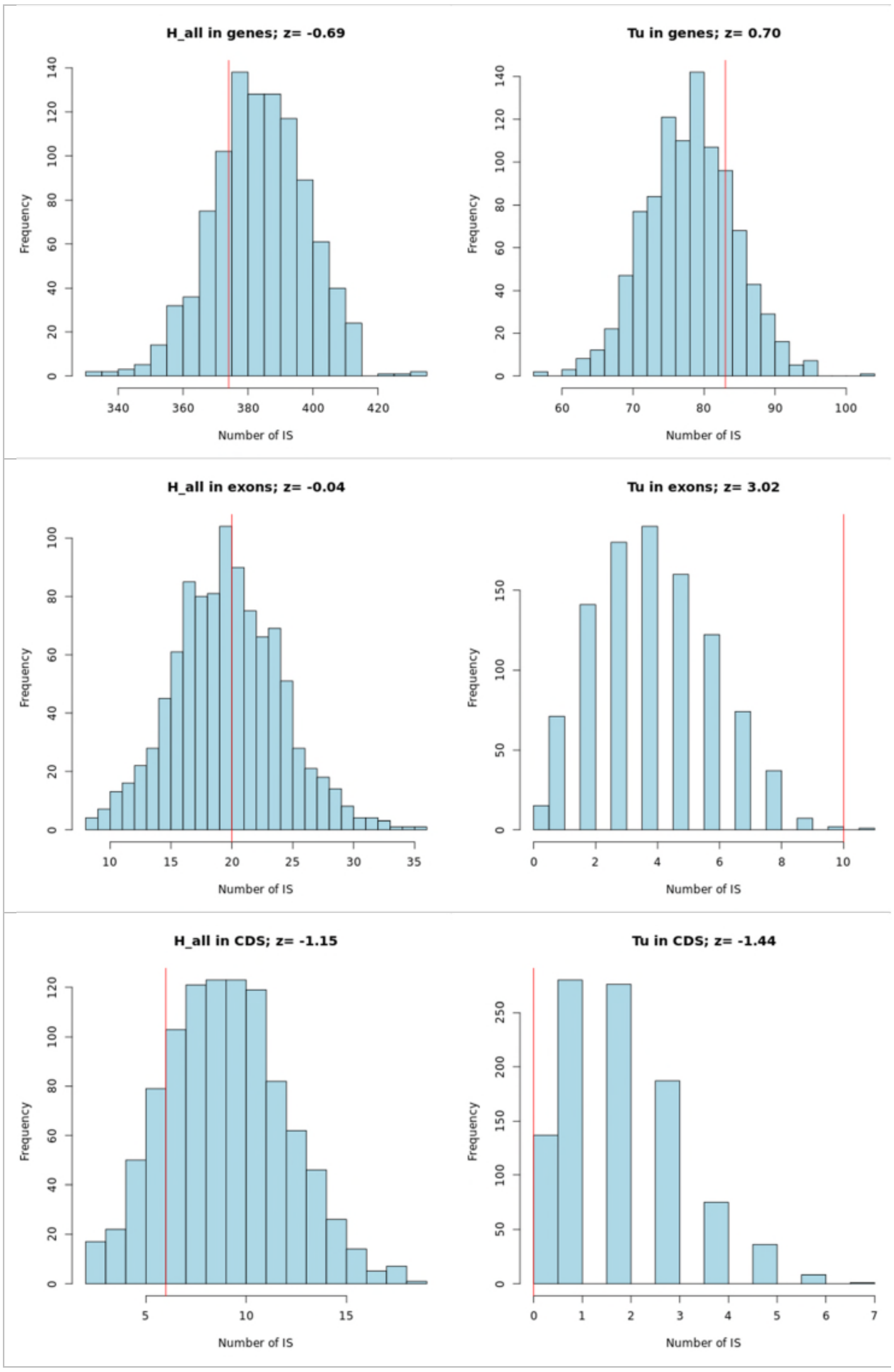

Supplementary Fig. 5| Genomic positions of integration sites compared to random distribution (1000 random shuffles). Red line indicates value in real data set and blue histogram represents the 1000 random shuffles. H_all = all IS in healthy tissue; Tu = tumour-specific IS; Z-scores of $-1.94 < z > 1.94$ are significant at $p < 0.05$. Only the number of tumour-specific IS in exons differs significantly from what is expected at random with $P = 0.002$.

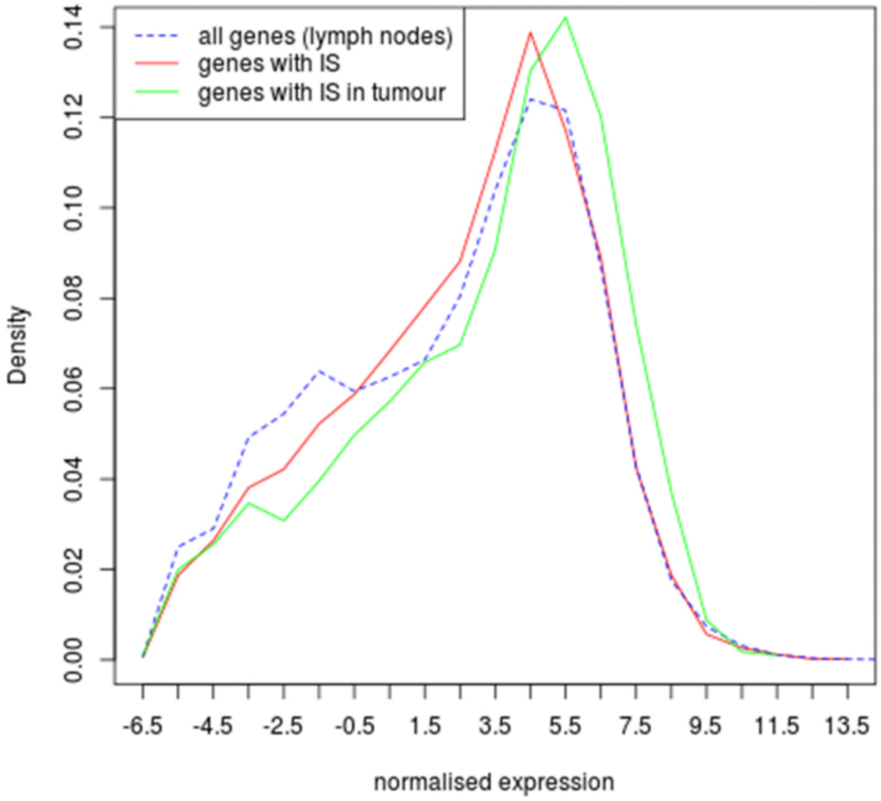

Supplementary Fig. 6| Expression levels in lymph nodes of all genes compared to genes proximal to tumour-specific IS or IS in healthy tissues. Expression data was taken from Sarker *et. al.*[5]. Genes for each category were selected from the normalised expression data and the distribution of expression plotted as a density curve. The difference between tumour-specific IS (green) and all genes with IS (red) was small but significant. We used a non-parametric Wilcoxon test to compare the two groups (genes with tumour-specific vs genes with IS), P = 0.00502

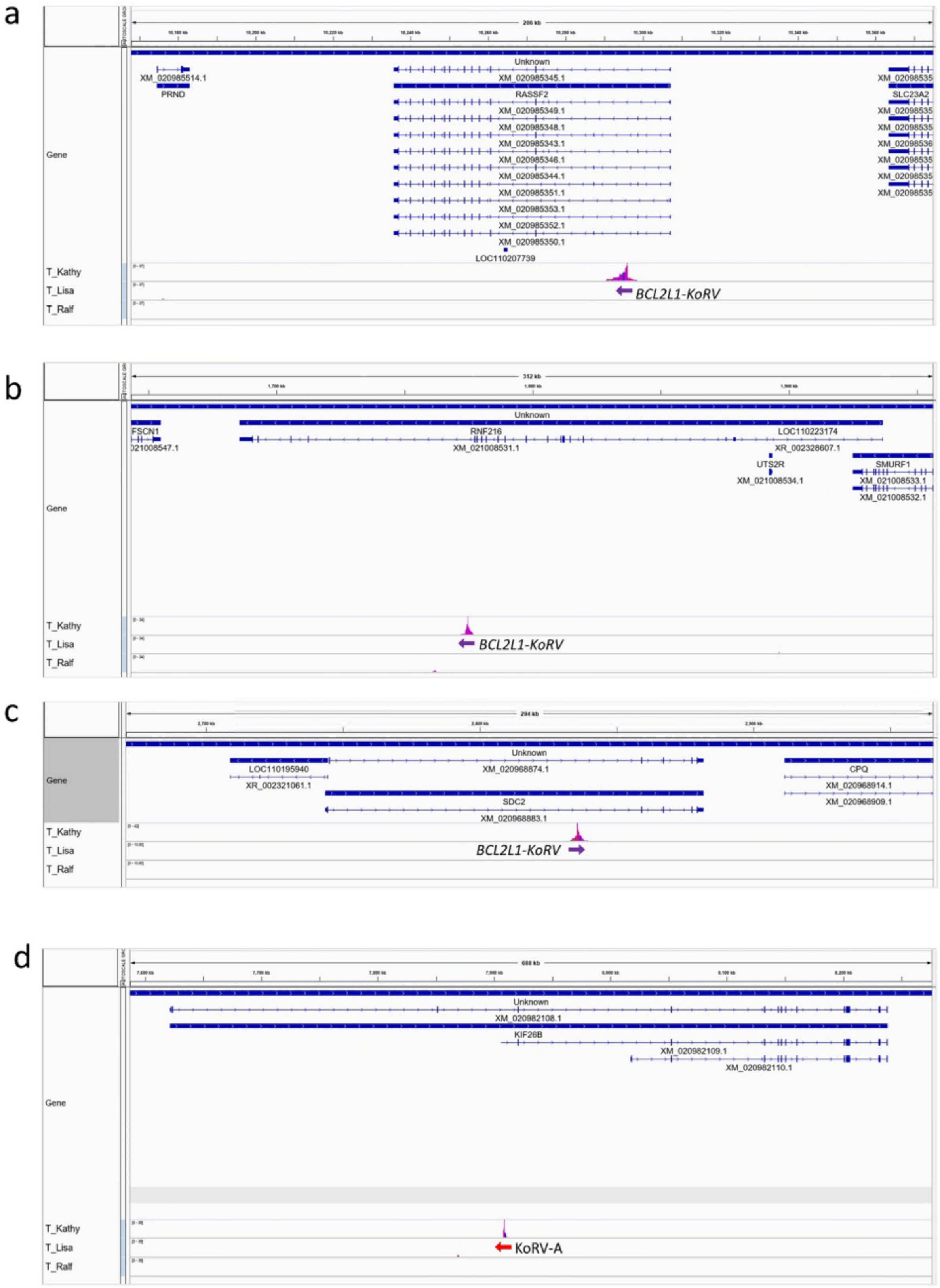
a
206 kb
Unknown
XM_020985514.1
PRND
XM_020985345.1
RASSF2
XM_020985349.1
XM_020985348.1
XM_020985343.1
XM_020985346.1
XM_020985344.1
XM_020985351.1
XM_020985353.1
XM_020985352.1
XM_020985350.1
LOC110207739
SLC23A2
Gene
T_Kathy
T_Lisa
T_Ralf
BCL2L1-KoRV
b
312 kb
Unknown
FSCN1
RNF216
LOC110223174
XM_021008531.1
XR_002328607.1
UTS2R
XM_021008534.1
SMURF1
XM_021008533.1
XM_021008532.1
Gene
T_Kathy
T_Lisa
T_Ralf
BCL2L1-KoRV
c
294 kb
Unknown
LOC110195940
XR_002321061.1
XM_020968874.1
CPQ
XM_020968914.1
XM_020968909.1
SDC2
XM_020968883.1
Gene
T_Kathy
T_Lisa
T_Ralf
BCL2L1-KoRV
d
688 kb
Unknown
XM_020982108.1
KIF26B
XM_020982109.1
XM_020982110.1
Gene
T_Kathy
T_Lisa
T_Ralf
KoRV-A

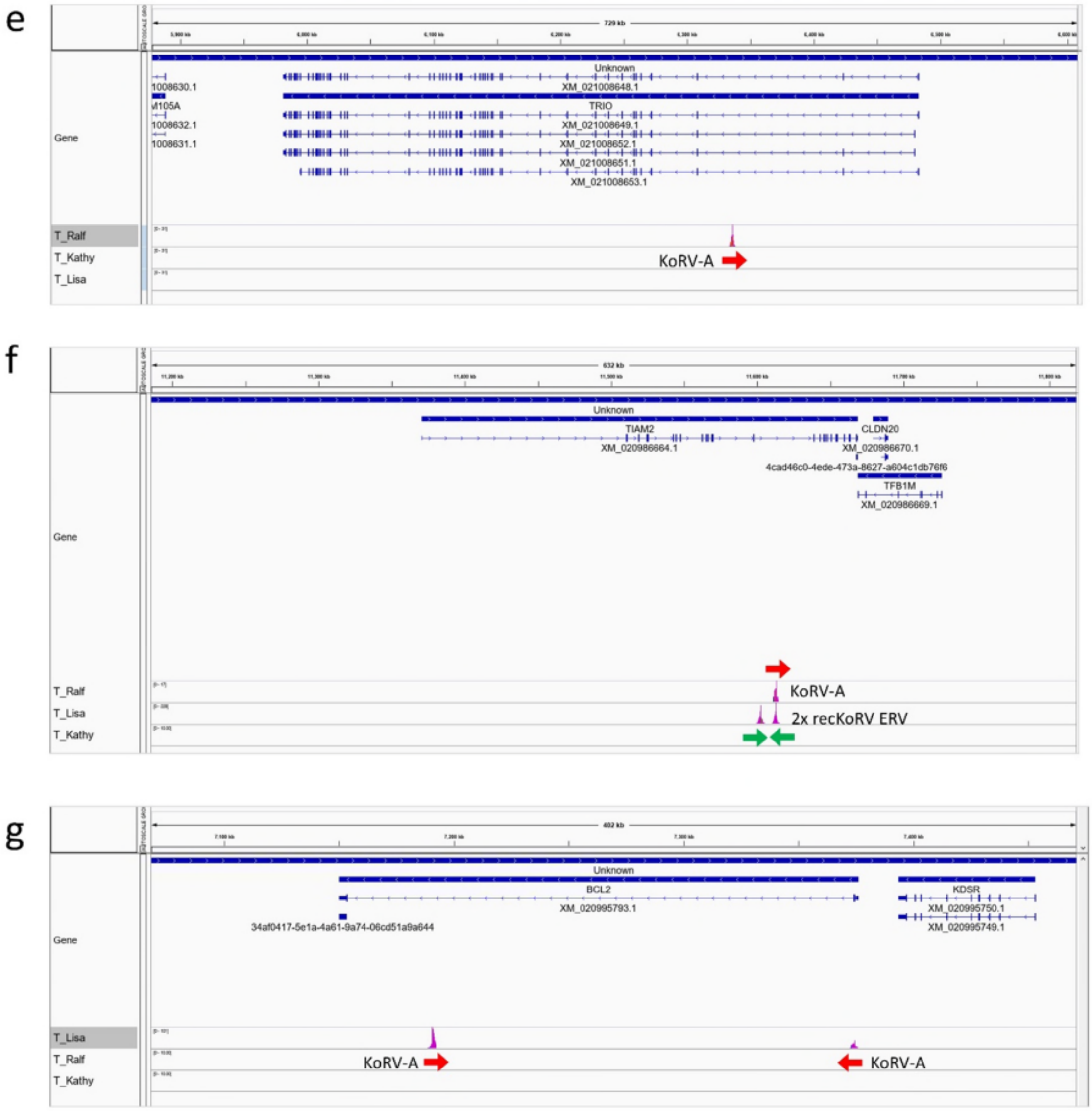

Supplementary Fig. 7| Relative positions and KoRV subtypes of IS in genes measeured for gene expression changes by qPCR. **a**. *RASSF2*, **b**. *RNF216*, **c**. *SDC2*, **d**. *KIF26B*, **e**. *TRIO*, **f**. *TIAM2*, **g**. *BCL2*. Genes are shown in blue and read coverage at tumour-specific IS in pink (peak coverage represents IS position). Arrows show the direction of the provirus (red for KoRV-A, purple for BCL2L1-KoRV, and green for recKoRV). *RASSF2*, *RNF216* and *SDC2* integrations (a, b, c) are all copies of the *BCL2L1* fusion (in the same orientation as the gene). *KIF216B*, *TRIO* and *TIAM2* (d, e, f) are all KoRV-A integrations in the opposite orientation to the gene. Lisa also has two recKoRV ERVs in *TIAM2* gene but the expression data indicates that these are not affecting expression as much as the tumour-specific KoRV integration in Ralf. Lisa has two KoRV-A integrations in *BCL2* (one in each orientation) but these don't lead to increased expression compared to tumour tissue in Ralf.

```
Kathy       8     TATTTGCATCCGGAGTTGTGTTCGTGTTGATCCTGGGAGGGTTTCTCAAGGT-GGAGGAC  66
LTR.1       422   ........................C...........................C.......  481

Kathy       67    TACCCGAA-ATTCGGGGTCCTACATTTGGGGGCTCGTCCGGGATTTGAGATTCCCACCCA  125
LTR.1       482   ........C...-......                                           499
inter1      1                              ...................C...............  35

Kathy       126   AGGACCGCCGAACCACCGACGGGAGGTAAGCTGGCCAGCAATCGCTCTATGTTCTCTCTG  185
inter1      36    .......................................G....................  95

Kathy       186   TGTCTGAATCTGAAAACTCTGACTGTCTATTCGGTGTGCGCGCATTTTTGGTTTCAGTTT  245
inter1      96    .......C..C.................................................  155

Kathy       246   ATTCCGGGTTAATCGATCTGAGATCGAGGTGTGAGTAGCGGACAGACGTGTTCGGGGGCT  305
inter1      156   ...................A.........C..............................  215

Kathy       306   CACTGCCCGGCAATCCTGGGAGACGTCCCAGGATC-GGGG-GGACCAGGGACGCCTGGTG  363
inter1      216   ...C...............................A....A...................  275

Kathy       364   AACCCTGCGGGTGAGGGATAAGACCCTCTCGGTATCGGTAGGCTCTTTTTCTTGTCTTTT  423
inter1      276   G....CA...CA-........                                         295

Kathy       424   TCGTGTGTGTCAATGCCGGCGTGTG-AGATTTGTCTGGGATGCAAAAGGAATGTTCCACC  482
ZBTB18      4250                       ....A..G....CT.......A....A.G..A..G..G.  4288

Kathy       483   TTCAGTAGTGTTGAACTCTGTGCCTAAGTTTTTGAATTACTTTTATTCTTCACTGTAATT  542
ZBTB18      4289  .....C...........G....TT...AAA.G.........G.....G.AT.........  4348

Kathy       543   GTCGACATGGGCTGGGGCgggggggggAGTCATGAAAAGTAAATATTGACGCAACAGAAA  602
ZBTB18      4349  .ATT.............-....                                        4369
ZBTB18      4409                            .....T...............A....T.......  4442

Kathy       603   TGCTATCTTTAGATGGCACCATTTGAAGAAACTGTTGAACATAAATGCACTGTTTTTATT  662
ZBTB18      4443  ...                                                           4445
ZBTB18      4561                                      ........................  4584

Kathy       663   TTATTTTCCCACTGTCTTAAAGTGGAAGGTGTTAGGTTGAATACAAATTAAGATACAAAT  722
ZBTB18      4606  ...C..............T....T....C.....A........A.......C........  4664
ZBTB18      4585  .......                                                       4591

Kathy       723   TGGTAGGTTGGCCACATAACAAGGAAAATGTGAGTGTTTATACTTGGCTTAAAGGTTTCC  782
ZBTB18      4665  ..A..AC..A..T................A.A.A......C.AA.......C..A...G.  4724

Kathy       783   ATGTGCGGTGTACATTTGTTACAAGCTTCTAATTCTACAGAACCCTCAGGTTTAATCGAt  842
ZBTB18      4725  ......A....G.....A.A....A.........GC.........                 4769

Kathy       843   ctctctctctctctTTATCCACACCGGTTTCACTGAAAAGTCGTGAGGTCAAGGGCTCAC  902
gag-pol     42                                   ......G....A.....A.A...........  72

Kathy       903   AATCTTTCAGTGGAGA-CGCAAAGGGAAAAATGGCAAATCCTCTGCTCCTCCGTGTGGCC  961
gag-pol     73    ........C.......TAAG......-...G.......C.T....T.......A......  131

Kathy       962   CACGTTCGAGGTGGGATGTCCACAGGAGGGGAACTTTTAATCCTTCTATTATTTCTGCAG  1021
gag-pol     132   .......A.A........G....C.......-............................  190

Kathy       1022  TCAAAGCGGTTGTCTTCC-GGAGACTGGAAGACACCCGGACTAGATTTCCTACACCCCCC  1080
gag-pol     191   .....AG.A.........A..........G...........C..G..C......        244
gag-pol     900                                                          .....  904

Kathy       1081  TTAATCAACTTCAGTGGGATCACAACTTGGCTGAAGGCTTTAGAGGTTGTGAGGACTCAG  1140
gag-pol     4727                                 .............................  4755
gag-pol     905   ......G..C................AC.........                         941

Kathy       1141  ATCTGGGACCAGATGAAAGAAGCCTGTAG-CCTGGTCCGGCAGCCTTGAGCCTCGGTGGA  1199
gag-pol     4756  ..............A...........AC..G......                         4791
gag-pol     4850                                   ...........................  4876

Kathy       1200  AAGGCTCATACCTGGTGCAACTGCTTCTCATCTCAAACCCGCGCCACTTAGGGCACACGA  1259
gag-pol     4877  .....C............T....                                       4899
env         2                          ..........................C.G......C...  40

Kathy       1260  TGAGTCTTGGGAACTGaaaaaaaaCTGATCATCCTCCTAATTTGCGTGTTCAGCAGCGCC  1319
env         41    ......C.........G....G-.............T...G..........G..G.....  99

Kathy       1320  GAAATGAACCCTCACCAACCCATGACTCCCACCTGGCAGAACCAGCATTTTTGCCCTGAA  1379
env         100   ........                                                      107
env         121         ......................T..........                       153
BCL2L1.U5   141                                   ........................  164

Kathy       1380  TCTCAGAAGAGAATTGTCAAGCAGATCCCAGACCCAGAGAGCAGGTGCTTTGGACAATGA  1439
BCL2L1.U5   165   ............................................................  224

Kathy       1440  ACTGGATCAGCCCATTCCTGATGTAAAAATGTCTCACAGCAACCGGGAGCTGGTGGTTGA  1499
BCL2L1.U5   225   ............................                                  252
BCL2L1.e1   546                               ................................  515

Kathy       1500  CTTTCTTTCTTACAAGCTCTCACAGAAAGGATACAGTTGGAGTCAATTTGAAGACGAGAA  1559
BCL2L1.e1   514   ......................................................T.....  455

Kathy       1560  TAGGACTGAGG-CCC-AGAAGGGACAGAAATACCTAGTACTGTGAATGGCAGCCCCTCTT  1617
BCL2L1.e1   454   ...........-...C............................................  396

Kathy       1618  GGCACCCTGCTGACAGCCATGCAGTGAGTGGGGCCACAGGACACAGCAGCAGCCTGGATG  1677
BCL2L1.e1   395   ............................................................  336
```

```
Kathy        1678  CCCATGAGACAATACCGGTGGCTGCTGTGAAGCAAGCTTTGAGGGAGGCAGGAGATGAAT  1737
BCL2L1.e1    335   ............................................................  276

Kathy        1738  TTGAACTCCGGTACCGAAGGGCCTTCAGTGACCTGACATCCCAGCTCCACATCACTCCAG  1797
BCL2L1.e1    275   ............................................................  216

Kathy        1798  GGACGGCTTATCAGAGCTTTGAGCAGGTAGTGAATGAACTCTTTCGGGATGGGGTGAACT  1857
BCL2L1.e1    215   ............................................................  156

Kathy        1858  GGGGCCGAATTGTGGCATTCTTCTCCTTCGGAGGGGCATTGTGTGTGGAAAGCGTGGATA  1917
BCL2L1.e1    155   ............................................................  96

Kathy        1918  AGGAGATGGAAGTCTTGGTAGGACGAATCACCTCCTGGATGGCCACTTACTTGGATGACC  1977
BCL2L1.e1    95    ............................................................  36

Kathy        1978  ACCTAGACCCTTGGATCCAAGAAAATGGCGGTTGGGACACCTTCGTGGAGCTTTATGGGA  2037
BCL2L1.e1    35    ...................................                           1
BCL2L1.e2    138                                      .........................  114

Kathy        2038  ACGATGCAGCTGCAGAGAGCCGGAAGGGCCAGGAACGCTTCAACCGATGGCTGCTGACTG  2097
BCL2L1.e2    113   ............................................................  54

Kathy        2098  GCATGACAGTGGCTGGTGTAGTCCTGCTGGGGTCCCTGTTCAGCCGGAAGTGACCATGCA  2157
BCL2L1.e2    53    .....................................................         1

Kathy        2158  CTGAATCTGCCTTGGAACCTCTCTGATCCACCCACTCCAACCCATCTTGGCCTGTACTCT  2217
BCL2L1.U3    944   ............................................................  1003

Kathy        2218  GTCCTGACACAGAGGAAATCTACTACATGCAGCTGATGTCTATCACATGGGGCTAAACCT  2277
BCL2L1.U3    1004  ............................................................  1063

Kathy        2278  GGCCTTCTTCTATGGTCCTAATCCCCTCCCTTTCTGAAGTGGTGCTCCAGAATAGGAGAG  2337
BCL2L1.U3    1064  ..............................                                1093
env          1577                          .......G........A...................  1612

Kathy        2338  GCCTTGATCTGCTATTTTTGAGGGAAGGGGGCCTTTGTGCAGCCCTAAAAGAGGAATGCT  2397
env          1613  .....................A......................................  1672

Kathy        2398  GTTTCTATGTTGACCACTCAGGCGCGGTGCGAGACTCCATGAGGAGACTCAAGGAAAGGT  2457
env          1673  ............................................................  1732

Kathy        2458  TGGATAAGAGGCAGTTAGAGCGCCAAAAGAATTTAAGTTGGTACGAGGGATGGTTCAACC  2517
env          1733  .A...................A......................................  1792

Kathy        2518  GTTCCCCCCGGCTTACTACTTTACTGTCTGCCCTTGCTGGTCCCCTGCTACTCCTCCTTC  2577
env          1793  ........T...................................................  1852

Kathy        2578  TGTTACTCACCCTCGGGCCTTGTGTCATCAATACGTTAGTGCAATTCATCAATGATAGGG  2637
env          1853  .................................A..........................  1912

Kathy        2638  TTAGTGCAGTAAGGATTCTGGTTCTCAGGCACAAGTACCAGACCCTAGACAACGAGGATA  2697
env          1913  ............................................................  1972

Kathy        2698  ACCTTTAATTCTGCTCTATGATTAGAGCTACCCTCAAAGAAAATGGGGGAATGAAGGAGG  2757
env          1973  ........                                                      1980
inter2       1             .........................................             41
LTR.2        1                                                      ...........  11

Kathy        2758  CAGAAATCATGAGGCAGAAATCATTCCGTGGAGTATGGAAACTACCCGGAGGGCCCAAGG  2817
LTR.2        12    ............................................................  71

Kathy        2818  TTTAGGGACAGGTGCAGCCAGGCACAGTAAAAGGTCAGAGCAAGAAAAACAAGGAAGATT  2877
LTR.2        72    ............................................................  131

Kathy        2878  TGGAGTGCCAAACA-G-ATATCTGTGGTCATGCACCTGAGTCCCCACCCCGGACTTAGGC  2935
LTR.2        132   .........G....G.-........................................T..  190

Kathy        2936  AAACAGTTCCCAGGGTGGAGTGCCAAACAGGATATCTGTGGTCATGCACCTGAGTCCCCA  2995
LTR.2        191   .....A.......                                                 203
LTR.2        132                  .........G...................................  176

Kathy        2996  CCCCGGACTTAGGCAAACAGTTCCCAGGGTGGAGTGCCAAACAGGATATCTGTGGTCATG  3055
LTR.2        132                                ..........G.....................  162
LTR.2        177   ...........T.......A.......                                   203

Kathy        3056  CACCTGAGTCCCCACCCCGGACTTAGGCAAACAGTTCCCAGAAACAGCTGAGCTCATAAC  3115
LTR.2        163   .........................T.......A..........T...............  222

Kathy        3116  AGTTTCTAGGGTGCCCCT  3133
LTR.2        223   ..................  240
```

Supplementary Fig. 8| Overview of *BCL2L1* gene transduction in Kathy. A single read from Kathy is shown, mapped to KoRV, *BCL2L1* and *ZBTB18*. The full coding sequence and partial UTRs of *BCL2L1* are flanked by the start and end of the KoRV *env* gene and fragments of the 3' UTR of *ZBTB18* are flanked by the KoRV *gag* gene. The KoRV genome is annotated according to its parts: LTR.1 = 5' LTR; LTR.2 = 3' UTR; inter1 & inter2 = intervening sequences between

the LTRs and the other KoRV genes (*gag-pol* and *env* respectively). For *BCL2L1*, U5 = 5' UTR, U3 = 3'UTR, e1 = exon 1, e2 = exon2.

a

Query = PacBio Read from Elise, with KoRV containing ZBTB18 :

```
Length=1594
                                                                      Score     E
Sequences producing significant alignments:                          (Bits)  Value

  inter1::KoRVA:505-969                                                455     4e-130
  LTR.1::KoRVA:0-505                                                   394     8e-112
  gag-pol::KoRVA:969-6033                                              217     2e-58
  ZBTB18                                                               189     4e-50


Query       428   AATTTGCATCCGGAGTTGTGTTCGTGTTGATCCTGGGAGGGTTTCTCAAGGTCGGAGGACTACCCGAA-A  496
LTR.1      423    .......................C...........................................C.  491

Query       497   TTCGGGTCCAACATTTGGGGGCTCGTCCGGGATCTGAGATTCCCACCCAAGGACCGCCGAACCACCGACG  566
LTR.1       492   ..G.....TTT...                                                          505
inter1      1                   ........................................................  56

Query       567   GGAGGTAAGCTGGCCAGCAATCGCTCTATGTTCTCTCTGTGTCTGAATCTGAAAACTCTGACTGTCTATT  636
inter1      57    ..................G...........................C..C....................  126

Query       637   CGGTGTGCGCGCATTTTTGGTACCAGTTTATTCCGGGTTAATCGATCTGAGATCGAGGTGTGAGTAGCGG  706
inter1      127   .....................TT.........................A.........C...........  196

Query       707   ACAGACGTGTTCGGGGGCTCACTGCCCGGCAATCCTGGGAGACGTCCCAGGATC-GGG--GGACCAGGGA  773
inter1      197   ......................C...............................A...GA..........  266

Query       774   CGCCTGGTGGACCCTGCGGGTGAGGGATAAGACCCTCTCGGTATCGGTAGGCTCTTTTTCTTGTCTTTTT  843
inter1      267   ...............CA...CA-.......                                          295
inter1      348                                                    .A.........C.........  368

Query       844   CGTGTGTGTCAATGCCGGCGTGTGAA-ATTTGTCTGGAATGCAAAAGGAATGTTCCACCTTCAGTAGTGT  912
ZBTB18      4250                      ......GG....CT...G...A....A.G..A..G..G......C.....  4299

Query       913   TGAACTCTGTGCCTAAGCTAT-TGAATTACTTTCATTCTTTACTGTAATTGTCGACATGGGCTGGGGCgg  981
ZBTB18      4300  ......G....TT...-AA..G.........G.T...G.A...........ATT.............-..  4367

Query       982   ggggggAGTCATGAAAAGTAAATATTGACGCAACAGAAATGCATGAATTTCAATATACTTTTATCTTTAG  1051
BCL2L1      4368  ..                                                                      4369
ZBTB18      4409       .....T...............A....T..............G..........T....G.....    4471

Query       1052  ATGGCACCATTTGAAGAAACTGGTATTATGAAGAACATAAATGCACTGTTTTTATTTTAT----------  1111
ZBTB18      4529  ..A............A........G...................................TTTATATAAT  4598

Query       1112  ----------TTTCCCACTGTCTTAAAGTGGAAGGTGTTAGGTTGAATACAAATTAAGATACAAATTGAT  1171
ZBTB18      4599  TTAAATCTAC..............T....T....C.....A........A........C...........  4668

Query       1172  AGGTTGGCCACATAACAAGGAAAATGTGAATGTTTCTAATTGGCTTAAAGATTTCCATGTGCAGTGTACA  1241
ZBTB18      4669  .AC..A..T................A.A.......AC..A........C.....G............G..  4738

Query       1242  TTTGTAACAGGCTTCTAATTCCACAGAACCCTCAGGCTTAATCGATCTCTCCCTCTTTATC-CACACTGG  1310
gag-pol     21                                  A.GG.A.AGGG.GAG..GAC......C.C..TT.....A.  40
ZBTB18      4739  ...A.....AA.........G..........                                         4769

Query       1311  TTTCACTGAAAAGTCGTGAGGTCAAGGGCTCACAATCTTTCAGTAGAG-TCGCAAAGAGAAAAAGGGCAA  1379
gag-pol     41    -A......G....A.....A.A...................C..G...A.AAG....-.G...GT.....  108

Query       1380  ACCCTCTGTTCCTCCGAGTGGCCCACGTTCGAGGTGGGATGTCCACAGGAGGGGAACTTTTAATCCTTCT  1449
gag-pol     109   ...T..........................A.A........G....C.......-...............  177

Query       1450  ATTATTTCTGCAGTCAAAGCGGTTGTCTTCC-GGAGACTGGAAGACACCCGGACTAGATTTCCT-AGTCC  1517
gag-pol     178   ..................AG.A.........A..........G...........C..G..C...-.      242
```

b

```
Query= sequenced PCR product in Elise using primers in ZBTB18 and BCL2L1.

Length=1302
                                                                      Score     E
Sequences producing significant alignments:                          (Bits)  Value

  gag-pol::KoRVA:969-6033                                             324     5e-91
  BCL2L1_mRNA XM_020990688.1 PREDICTED: Phascolarctos cinereus BC...  154     7e-40
  ZBTB18                                                              75.0    6e-16
  inter1::KoRVA:505-969                                               27.0    0.16


Query       68    TGTCTTAAAGATTTCCATGTGCAGTGTGCATTTGTAACAGGCTTCTAATTCCACAGAACCCTCAGGTTTA  137
gag-pol     7                                                                   ....G.G.  14
ZBTB18      4709  ..G.....C.....G..................A.....AA.........G..........          4769

Query       138   ATCGATCTCTCTCTCTCTATCCACACTGGTTTCACAGAAAAGTCGTGAGGTCAAGGGCTCACAATCTTTC  207
gag-pol     15    G....C.C..........-.--.....A.-A....T.G....A.....A.A..................  80

Query       208   AGT--AGAGACGCAAAGAGAAAAAGGGCAAACCCTCTGTTCCTCCGAGTGGCCCACGTTCGAGGTGGGAT  275
gag-pol     81    C..GG...T.A.-....-.G...GT........T.........................A.A.......  148

Query       276   GCCCACAGGAGGGGAACTTTTAATCCTTCTATTATTTCTGCAGTCAAAGCGGTTGTCTTCC-GGAGACTG  344
gag-pol     149   .G....C.......-.................................AG.A.........A........  217

Query       345   GAAGACACCCGGACTAGATTTCCTACACCGTGATGTGACAGGACCCCCCCTTAATCAACCCCAGTGGGA  414
gag-pol     218   ..G...........C..G..C......                                            244
gag-pol     900                                                ...........G...T........  923

Query       415   TCACAACTTGGCTGAAGG-AGAGAAACGTCGCCTAGTCTACCACCGGACTCTAGTGGCGGGTCTCAACGG  483
gag-pol     924   .......AC.........T..-.G.G....T...G.......G......................A..  992

Query       484   GGCAAACAGGCGTCCCACCAATTTGGCCAAGGTAAGAGAAGACTTGCAGGGCCCAATAGAACCCCCGTCC  553
gag-pol     993   ....GCA.....C..........................T.........A....CC............  1062

Query       554   GTTTTTTTAGAACGTCTAATGGAGGCTTATAGGAGATATACCCCATTTGATCCCTCTTCTGAGGGACAAA  623
gag-pol     1063  ......C...............................................................  1132

Query       624   AGGCGTGCCATGTAGGTTCAGTGAGAGCACCGGCTCATGCGTCCTAACCTGCAGCCCGCGGCGAGGGGGC  693
gag-pol     1133  .....                                                                  1137

Query       694   GACCCGCGCGCCCACACCCTCATGTCTATACGAGTAACCACCCATTTTTCCCCTGCACTCTCAGAAGAGA  763
BCL2L1      144                                      .....-G.......G.....-.A............  176

Query       764   ATTTTCCAAGCAGATCCCACACTCAGAAAACGAGATGCTATTGGGGCAATATACCCCTATCAGCCCTCTT  833
BCL2L1      177   ...G.-.............G..C....G.G.-..G....-....A-....GA..TGG-........A-..  240

Query       834   CTTGATGTACAAAGAATCTCACACCAACCGGGCAGCTGGTGGTTTGACTTTTCTTTTTTTACAAGCTGCT  903
BCL2L1      241   .C.......A...TG-.......G........-...........-......-....C..-........-..  304

Query       904   CACAGTAAAGGATACAGCTTGGAGTGCACTTTGTAAGAACCAGAATAAGAAAAGACGGCCCCGAAAAGGA  973
BCL2L1      305   .....-............-.......-..A....-....TG-......G..CT-..-.......-.G....-  365

Query       974   GAAA-AAATACC  984
BCL2L1      366   ..C.G.......  377
```

Supplementary Fig. 9| Overview of *ZBTB18* containing sequences in Elise. a, A small number of eads were found in the PacBio data from Elise that contained *ZBTB18* joined to gag-pol in a similar way to what was seen in Kathy. An example is given showing the alignment to KoRV and *ZBTB18*. No reads containing *BCL2L1* were detected in Elise. A PCR was carried out using primers from *ZBTB18* and *BCL2L1* producing a faint band, which was subsequently Sanger sequenced. **b**, Alignment of Sanger sequencing product from PCR in Elise. The product contains sequence from *ZBTB18* and *BCL2L1* but the junctions are not the same as those in Kathy, and the *BCL2L1* sequence contains many mismatches, unlike the *BCL2L1* sequence in

Kathy which contains almost no mismatches. It is unclear if the sequences from Elise and Kathy are related but it seems most likely that they arose independently.

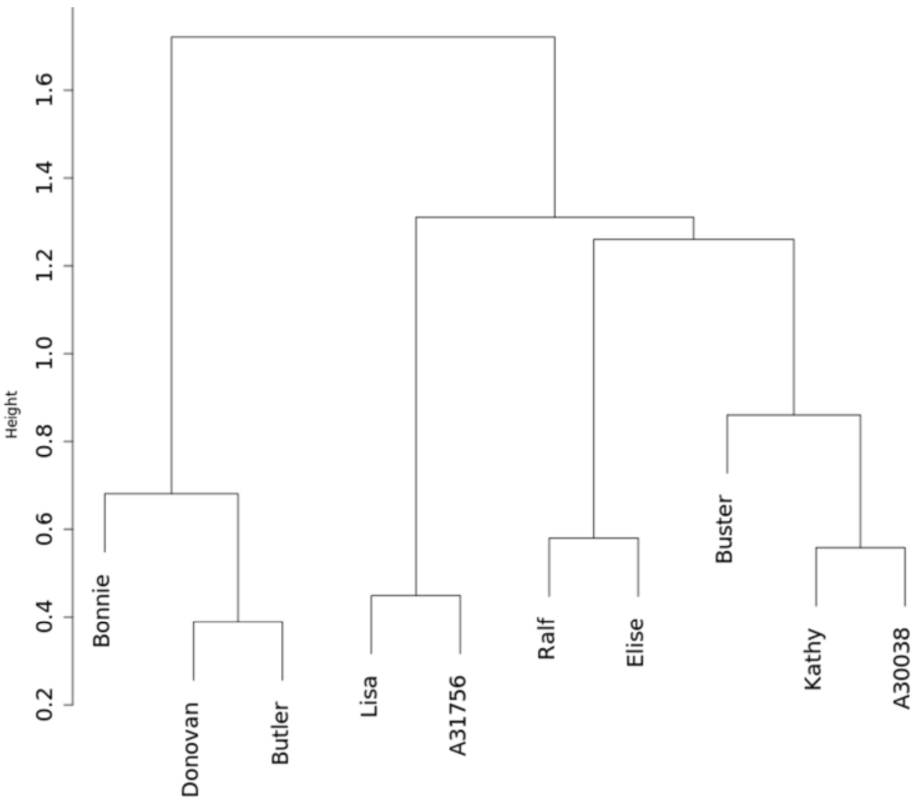

Supplementary Fig. 10| Dendrogram of KoRV IS showing relationships between koalas. Clustering was performed using *hclust* in R with a binary distance matrix. Analysis of shared IS was carried out blind, before metadata of locations were given.

a.

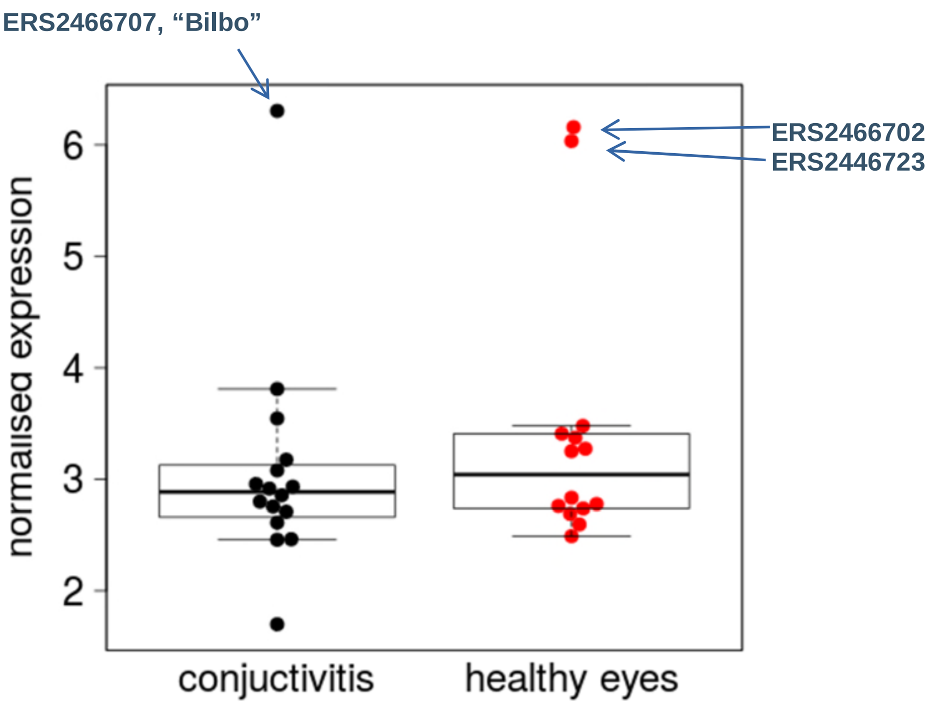
ERS2466707, "Bilbo"
ERS2466702
ERS2446723
normalised expression
6
5
4
3
2
conjuctivitis
healthy eyes

b.

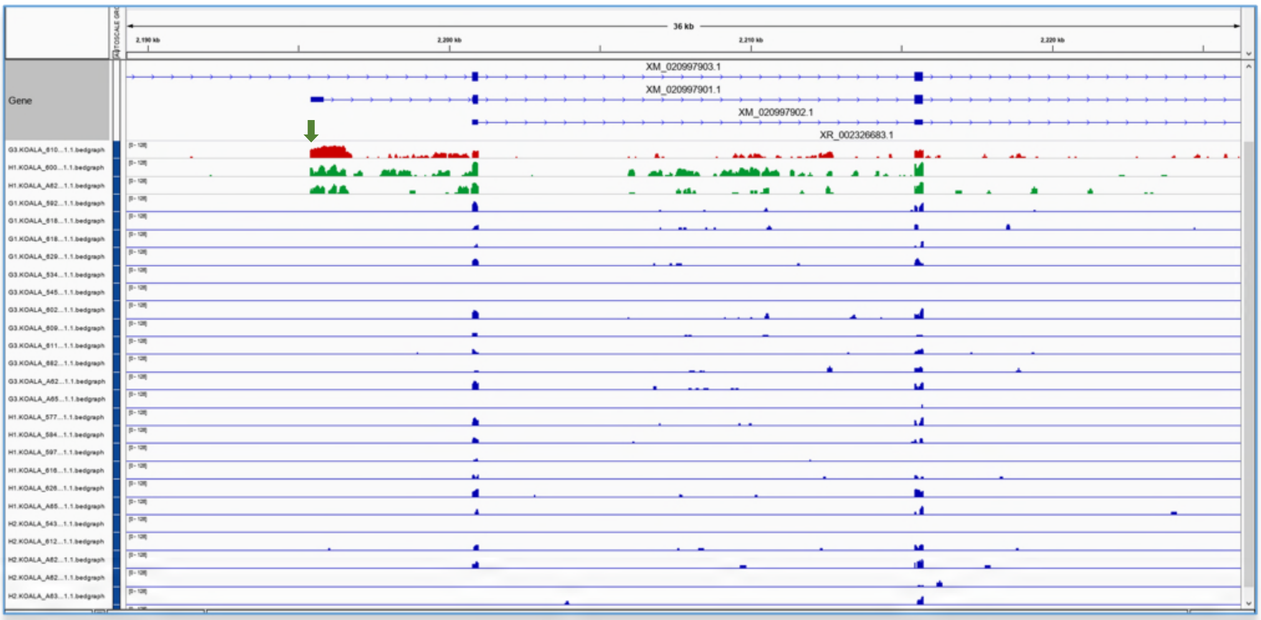
36 kb
XM_020997903.1
XM_020997901.1
XM_020997902.1
XR_002326683.1
Gene

Supplementary Fig. 11| IS in *ZFAT* 3'UTR. An IS was found in the 3' UTR of the gene *ZFAT* Ralf and Elise. The reference genome (Bilbo) also contains this IS. RNA-seq data exists for Bilbo in a study of chlamydial eye disease (PRJEB26467). Using this data, we looked at the gene expression around the IS in *ZFAT* in Bilbo and 29 other koalas. The data was normalised using the Limma package in R with sex and eye disease included as factors. Boxplots indicate the interquartile range (IQR) with the centre line showing the median; whiskers show 1.5 × IQR. n = 30. **a**, Three samples (one with chlamydial conjunctivitis and two with healthy eyes) had markedly increased expression in *ZFAT* (~5 – 8 fold higher). The three samples were:

Accession no.	Clinical description
1. ERS2466707	Bilateral Conjunctivitis. Reproductive disease. Also used by Australia museum for genetic tissue harvest study [= *Bilbo, reference genome*]
2. ERS2466702	Extremely emaciated; Leukaemia case
3. ERS2466723	Injured in vehicle collision. Eyes clear, cysitis = 0. Body score 3 [*out of 10*].

All 3 koalas were collected within a ~ 24 km radius of Brisbane city centre, in the area containing Ralf and Elise (all 8 other koalas in our study are from outside this radius). ERS2466702 was the only one of the 30 koalas in this study to be described as a leukaemia case.

b, The increase in gene expression was found to start exactly at the position of the IS. A screen shot of the region (produced using the Integrative Genomics Viewer[4]) shows the coverage (normalised and shown on a log scale) in each koala in *ZFAT.* The orange arrow indicates the position of the IS. The red track is Bilbo and the green tracks are the two other koalas with healthy eyes and high *ZFAT* expression. All other koalas are shown in blue. Increased expression for the three koalas is found in all subsequent exons of *ZFAT* [not shown].

a

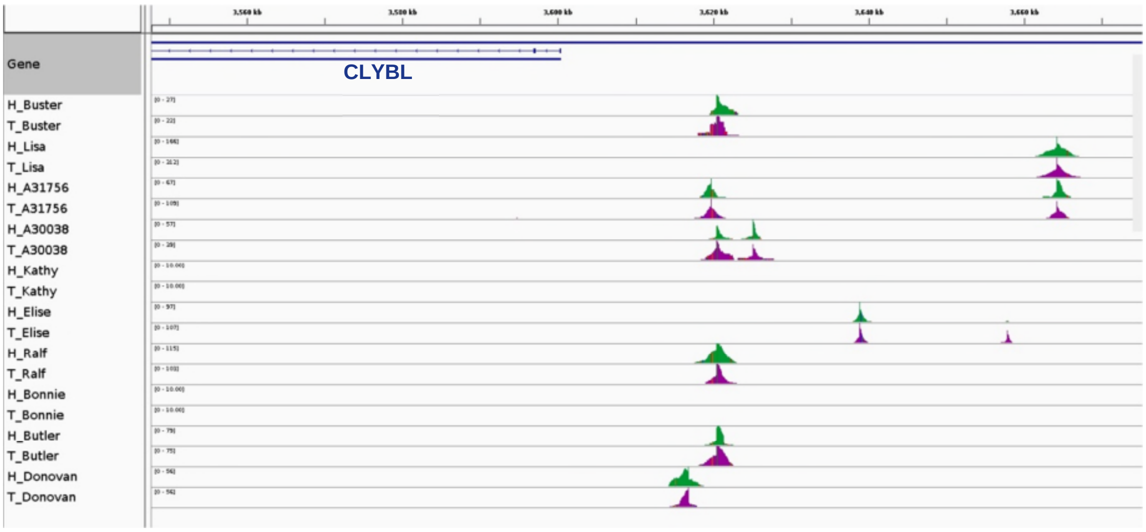
Gene
CLYBL
H_Buster
T_Buster
H_Lisa
T_Lisa
H_A31756
T_A31756
H_A30038
T_A30038
H_Kathy
T_Kathy
H_Elise
T_Elise
H_Ralf
T_Ralf
H_Bonnie
T_Bonnie
H_Butler
T_Butler
H_Donovan
T_Donovan

b

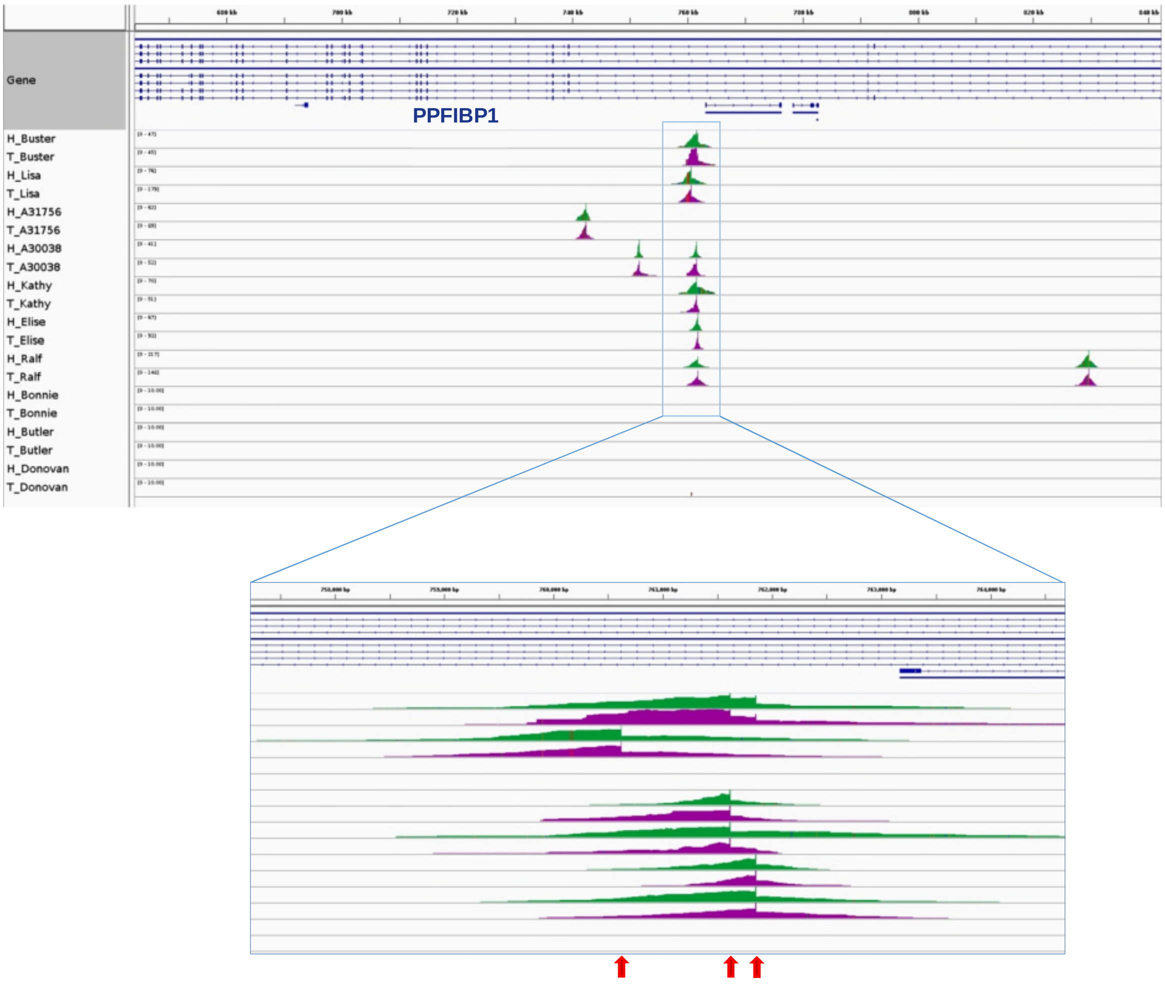
Gene
PPFIBP1
H_Buster
T_Buster
H_Lisa
T_Lisa
H_A31756
T_A31756
H_A30038
T_A30038
H_Kathy
T_Kathy
H_Elise
T_Elise
H_Ralf
T_Ralf
H_Bonnie
T_Bonnie
H_Butler
T_Butler
H_Donovan
T_Donovan

c

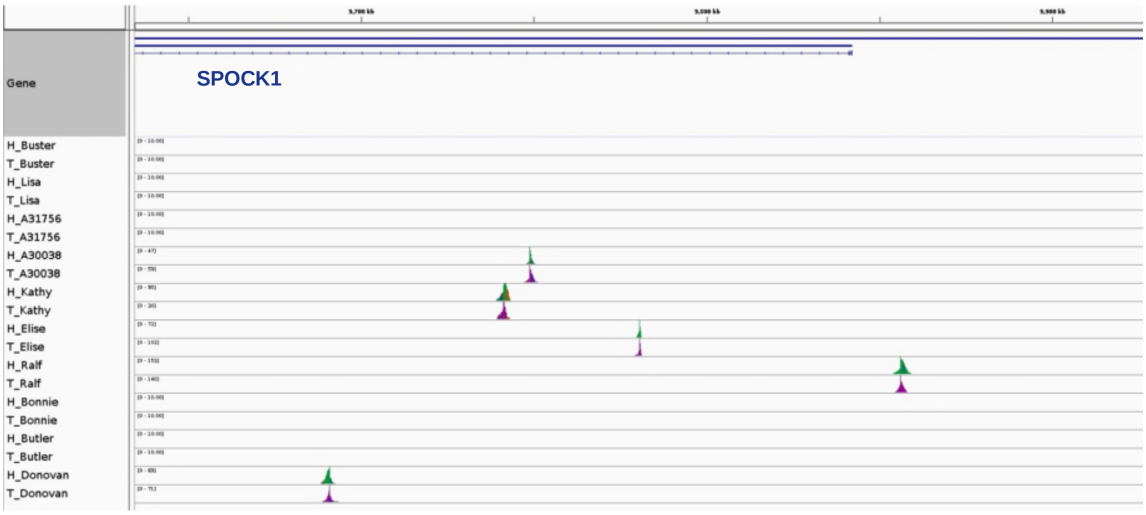

Supplementary Fig. 12| Examples of hotspot IS coverage in individual koalas. a, In the region directly upstream of the gene *CLYBL*, seven distinct IS were detected in a 47 kb region and 8 koalas had at least one IS in this region. Five IS were koala specific and 2 were shared. **b,** Six distinct IS were detected in a 87 kb region in an intron of *PPFIBP1* in 7 koalas. Three of these IS are located within 1.2 kb of each other (shown in sub-panel with IS marked by red arrows) and are directly upstream of an ncRNA. **c,** Five IS within a 165 kb region in *SPOCK1* in 5 koalas, none of which are shared. Alignment coverage from healthy and tumour tissues are shown in green and purple respectively. Koalas are designated with H and T before their names to indicate healthy and tumour tissues. Most of the these IS are found in both healthy and tumour tissue in each koala.

Batch	Koala	Sex	Age	DOB	Date of sample (MM/YY)	Location	Diagnosis	M/B*	Healthy tissue	Tumour tissue
1	**Elise** (A525346)	F	7	1998	11/05	Lone Pine, QLD	**Lymphoma/leukaemia** (lymph nodes, liver, spleen, kidney, pancreas, heart)	M	Kidney	Tumour (tissue unknown)
1	**A31756**	M	11	1997	05/08	Kallangur, QLD	[*no pathology report*] Tumour in mesentery lymph (> 50g), pancreas, right adrenal, prostate	M	Kidney	Tumour (tissue unknown)
1	**A30038**	F	11	1997	02/09	Wivenhoe Pocket, QLD	[*no pathology report*] Mammary tumour, liver, enlarged lymph nodes	M	Lymph node	Mammary
1	**Bonnie** (A47813)	F	6	2007	06/13	Tyagarah, NSW	**Rhabdomyoma** (retrobulbar)	B	Kidney	Rhabdomyoma
1/2	**Ralf** (A47628)	M	6	2007	05/13	Thornlands, QLD	**Lymphoma/leukaemia** (lymph nodes, liver, spleen)	M	Colon	Lymph node
2	**Butler** (A54030)	M	3	2011	06/14	Tyagarah, NSW	**Fibrosarcoma** (shoulder, metastatic to lung)	M	Liver	Fibrosarcoma
2	**Kathy** (A55125)	F	5	2009	08/14	Lake Clarendon, QLD	**Anaplastic carcinoma** (thyroid origin?, lymph nodes, liver, lung, kidney)	M	Skeletal muscle	Liver
2	**Lisa** (A57620)	F	6	2007/ 08	12/14	Morayfield, QLD	**Lymphoma** (mesentery, omentum, heart, mammary, neck)	M	Hair	Mesentery
2	**Donovan** (A59491)	M	4	2011	05/15	Tyagarah, NSW	**Osteochondroma** (skull)	B	Kidney	Osteochondroma
2	**Buster** (A61069)	M	9	2006	08/15	Doonan, QLD	**Osteochondroma** (skull)	B	Liver	Osteochondroma

*M/B = Metastatic/Benign

Supplementary Table 1| Detailed list of koala samples ordered by sample collection date. Koala sample collection dates range over 10 years from 2005 to 2015. Tissue types for tumour and healthy tissue samples vary between koalas. Samples were sequenced in two batches (with one koala, Ralf, in both batches). QLD = Queensland; NSW = New South Wales. *M/B = metastatic/benign tumour.

Koala	Tissue	Batch	Seq Rep	Total reads	Mean length (bp)	s.d.
A30038	H	D1	1	4778	1258.3	625.2
A30038	H	D1	2	11399	467.9	314.2
A31756	H	D1	1	18854	1243.8	577.9
A31756	H	D1	2	35178	1021.4	584.7
Bonnie	H	D1	1	19881	1321.7	621.5
Bonnie	H	D1	2	33975	1101.2	597.2
Butler	H	A1	1	54980	1226.7	694.2
Buster	H	A1	2	22664	2056.7	940.3
Donovan	H	A1	1	46257	1441.1	781.4
Elise	H	D1	1	14040	1217.0	619.8
Elise	H	D1	2	8865	567.0	444.6
Kathy	H	A1	1	58905	1969.3	941.1
Lisa	H	A1	1	52587	1976.1	1095.0
Ralf	H	A1	1	57101	1935.5	996.2
Ralf	H	D1	2	12888	1589.0	744.2
Ralf	H	D1	3	23752	1588.0	769.7
A30038	T	D1	1	7522	1384.3	697.2
A30038	T	D1	2	17387	1269.0	668.4
A31756	T	D1	1	20028	1233.6	544.6
A31756	T	D1	2	35066	1056.7	584.3
Bonnie	T	D1	1	14839	1219.1	541.3
Bonnie	T	D1	2	10287	980.3	645.9
Buster	T	A1	1	45618	2177.7	1102.3
Butler	T	A1	1	30739	2007.5	852.3
Donovan	T	A1	1	52962	1174.8	740.0
Elise	T	A2	1	27708	527.7	367.2
Elise	T	D1	2	6602	982.9	454.3
Elise	T	D1	3	9331	424.2	263.1
Kathy	T	A1	1	21503	1355.6	1092.4
Lisa	T	A1	1	49801	1140.1	671.8
Lisa	T	A1	2	56470	2020.7	907.3
Ralf	T	D1	1	8424	1737.9	710.1
Ralf	T	D1	2	33869	1423.4	686.3

Supplementary Table 2| Overview of samples and read lengths. Koala samples were prepared and sequenced in two batches (A1 & D1), with Elise tumour tissue being additionally re-sequenced (A2). Total reads sequenced, their mean length (bp) and standard deviation (s.d.) is shown. Elise and A30038 had shorter average lengths than the other koalas, possibly due to degradation of DNA since these were two of the earliest collected samples. H= healthy; T= tumour.

Koala	Sex	Age	Diagnosis	Total IS	Both tissues	Tumour specific	Healthy specific	Shared ERVs
**Lisa**	F	6	Lymphoma	172	113	57	2	38
**Ralf**	M	6	Lymphoma/leukaemia	147	110	37	0	31
**Kathy**	F	5	Anaplastic carcinoma	144	106	24	14	32
**A31756**	M	11	Tumour in mesentery lymph (> 50g)	139	94	41	4	36
**Elise**	F	7	Lymphoma/leukaemia	125	117	4	4	36
**A30038**	F	11	Mammary tumour	122	117	0	5	28
**Donovan**	M	4	Osteochondroma (skull)*	96	91	4	1	60
**Butler**	M	3	Fibrosarcoma (shoulder)	93	88	1	4	55
**Buster**	M	9	Osteochondroma (skull)*	95	88	4	3	17
**Bonnie**	F	6	Rhabdomyoma (retrobulbar)*	89	89	0	0	45

* Benign tumour

Supplementary Table 3| Number of IS per koala. Total number of IS identified, and the number in both tumour and healthy tissues (ERVs) or specific to one tissue are given for each koala. The number of ERVs shared with other koalas is also given. Koalas affected by leukaemia/lymphoma harbour more IS in general than those with non-haemopoietic tumours (osteochondroma, fibrosarcoma, rhabdomyoma) and have more tumour-specific IS. Koalas Elise and A30038 have many IS in healthy tissue and few or no tumour-specific IS; it is possible that in these samples the healthy tissue contains some metastasised cells from the tumour tissue as the cancers had progressed.

	B	D	F	G	H	I	% reads with env	% variants
**Ralf**	0	35	0	0	0	0	0.663	0.006
**Elsie**	0	0	0	0	0	0	0.001	0.000
**Bonnie**	0	0	2	0	0	0	0.001	0.001
**Butler**	10	0	251	0	0	0	1.839	0.076
**Donovan**	0	0	12	0	0	0	0.125	0.003
**Kathy**	5	11	0	0	0	0	0.372	0.005
**A30038**	0	0	0	0	0	0	0.003	0.000
**Lisa**	0	65	0	162	0	0	0.365	0.036
**A31756**	0	15	0	25	0	0	0.030	0.009
**Buster**	0	0	0	0	0	0	3.142	0.000

Supplementary Table 4| KoRV subtypes. There are at least 9 known subtypes of KoRV (KoRV-A through KoRV-I; KoRV-J is now classified as a strain of KoRV-B). KoRV-A is the main subtype, found in 100% of northern Australian koalas and in all zoo koalas derived from northern Australia (which accounts for the majority of zoo koalas worldwide). It is also the most highly expressed when viral load is measured. KoRV-B has been found to use a different membrane receptor than KoRV-A for infection and is thought to be exogenous. KoRV-B was isolated from koalas with malignant neoplasms at San Diego Zoo[6]. In addition to KoRV-A and KoRV-B, seven other putative subtypes have been described on the basis of variation in the envelope gene. We looked for KoRV subtypes in our 10 koalas using the highly variable envelope region[7]. To cross the variable envelope region, a read must extend for 2,244 bp from the 3’ end of the IS, and a small proportion of reads are long enough to cover this region. Two koalas, Kathy and Butler, were found to be positive for KoRV-B. Koalas were otherwise found to be positive for KoRV variants (D, F and G). The same variants were generally within related groups of koalas (see main text Fig 3 and Supplementary Fig 9 for groups; groups are defined by colour in the above table): Lisa and A31756 had reads matching to KoRV-D and -G; Butler and Donovan and Bonnie had KoRV-F; and Ralf had KoRV-D (Elise had one 50 bp hit to KoRV-D). Elise, Bonnie and A30038 had almost no reads long enough to cover the variant envelope region to identify subtypes and so they had extremely few or no hits (marked in grey). Buster had many reads coving the envelope region but no variants were detected. These subtypes were found at very low average coverage (0 – 0.076 % of reads) indicating that they are exogenous and only in a small proportion of cells, consistent with prior results on KoRV variants[8,9].

	Koala	Position	Gene	Gene Description
**Tumour-specific IS**	Ralf	Exon (*not annotated as UTR or CDS*)	*LYPD6B*	Believed to act as a modulator of nicotinic acetylcholine receptors (nAChRs) activity
	Lisa	5’ UTR	*IL6R*	Subunit of the interleukin 6 receptor, Dysregulated production implicated in the pathogenesis of many diseases, such as multiple myeloma, autoimmune diseases and prostate cancer
	Lisa	5’ UTR	*BCL2*	Blocks the apoptotic death of some cells such as lymphocytes. Associated with lymphoma.
	A31756	5’ UTR	*KRT13*	Keratin 13. KRT13 expression was elevated in bone, brain, and soft tissue metastatic prostate cancer cell lines
	A31756	3’ UTR	*CCNJL*	Cyclin-J-Like Protein
	Kathy	5’ UTR alt trans	*M1AP*	Meiosis 1 arrest protein
	A31756	5’ UTR alt trans	*TRAF4*	Plays a role in the activation of NF-kappa-B and JNK, and in the regulation of cell survival and apoptosis.
	A31756	lncRNA	*LOC110213370*	
	Lisa	lncRNA	*LOC110204142*	
	Ralf	pseudogene	*LOC110208445*	
**Healthy IS**	A31756	CDS	*CCHCR1*	Thought to act as a regulator of mRNA metabolism
	Lisa	CDS	*CDC23*	Cell Division Cycle 23. controls progression through mitosis and the G1 phase of the cell cycle.
	Lisa	CDS	*GNA15*	Decreased expression of GNA15 associated with inhibition of proliferation, activation of apoptosis in neuroendocine neoplasia
	Ralf	CDS	*METTL11B*	Methyltransferase Like 11B
	Buster	CDS	*PODNL1*	Highly expressed in mineralized tissues and in osteoblastic cells
	Elise	CDS	*RAB3IL1*	Encodes a guanine nucleotide exchange factor for the ras-related protein Rab3A
	Buster	3’ UTR	*GPR153*	
	Elise	3’ UTR	*LOC110208870*	
	Buster	5’ UTR	*LOC110214204*	
	Kathy	lncRNA	*LOC110218575*	
	A30038	ncRNA	*LOC110202241*	
**Shared in groups**	Ralf/Elise	3’ UTR	*HMBOX1*	Positively regulates telomere elongation.
	Ralf/Elise	5’ UTR alt trans	*ZFAT*	Transcriptional regulator involved in apoptosis and cell survival
	Donovan/Bonnie/Butler	3’ UTR alt trans	*LSAMP*	May function as tumour supressor
	Kathy/A30038	5’ UTR alt trans	*TMEM65*	May play an important role in cardiac development and function
	Donovan/Bonnie/Butler	lncRNA	*LOC110210102*	
	Kathy/A30038	pseudogene	*LOC110198350*	
**Shared, other**	Lisa/A31756/Kathy	ncRNA	*LOC110223184*	
	Elise/Bonnie	pseudogene	*LOC110220304*	

Supplementary Table 5| IS located in exons.

a. KEGG 2019

Tumour unique				
Term	Overlap	Adjusted P-value	Odds Ratio	Genes
Pathways in cancer	12/530	**0.006**	4.35	CDK6; TRAF4; CDH1; IL15; MYC; NCOA3; PLCG2; BCL2; COL4A5; IL6R; RUNX1; RASGRP3
Small cell lung cancer	5/93	**0.019**	10.34	CDK6; TRAF4; MYC; BCL2; COL4A5
Ras signaling pathway	7/232	**0.022**	5.80	SYNGAP1; RASA4B; RASA1; PLA2G10; PLCG2; EXOC2; RASGRP3
cGMP-PKG signaling pathway	4/166	0.310	4.63	ADORA3; KCNMA1; NFATC2; SLC8A1
Kaposi sarcoma-associated herpesvirus infection	4/186	0.312	4.14	CDK6; MYC; PLCG2; NFATC2
JAK-STAT signaling pathway	4/162	0.314	4.75	IL15; MYC; BCL2; IL6R
Chronic myeloid leukemia	3/76	0.321	7.59	CDK6; MYC; RUNX1
Primary immunodeficiency	2/37	0.325	10.40	CD8A; AICDA
Bladder cancer	2/41	0.329	9.38	CDH1; MYC
Th17 cell differentiation	3/107	0.333	5.39	NFATC2; IL6R; RUNX1

Healthy				
Term	Overlap	Adjusted P-value	Odds Ratio	Genes
Arrhythmogenic right ventricular cardiomyopathy (ARVC)	7/72	0.081	5.56	RYR2; DES; ITGB5; LAMA2; CTNNA3; CACNA1C; SLC8A1
Dilated cardiomyopathy (DCM)	7/91	0.084	4.40	RYR2; DES; ITGB5; LAMA2; MYL3; CACNA1C; SLC8A1
Non-small cell lung cancer	6/66	0.106	5.19	EML4; EGF; PRKCA; BRAF; PIK3R1; FHIT
Hypertrophic cardiomyopathy (HCM)	7/85	0.112	4.71	RYR2; DES; ITGB5; LAMA2; MYL3; CACNA1C; SLC8A1
Sphingolipid signaling pathway	7/119	0.153	3.36	PPP2R2B; SMPD1; SPHK1; PRKCA; PPP2R3B; PIK3R1; GAB2
Adrenergic signaling in cardiomyocytes	8/145	0.157	3.15	PPP1CC; RYR2; PPP2R2B; MYL3; PRKCA; PPP2R3B; CACNA1C; SLC8A1
Gastric cancer	8/149	0.164	3.07	SHC4; FGF7; EGF; MYC; BRAF; CTNNA3; PIK3R1; WNT2
ErbB signaling pathway	6/85	0.166	4.03	SHC4; EGF; MYC; PRKCA; BRAF; PIK3R1
Focal adhesion	10/199	0.169	2.87	SHC4; PPP1CC; CCND3; RELN; ITGB5; LAMA2; EGF; PRKCA; BRAF; PIK3R1
Endometrial cancer	5/58	0.176	4.93	EGF; MYC; CTNNA3; BRAF; PIK3R1

b. OMIM disease

Tumour unique				
Term	Overlap	Adjusted P-value	Odds Ratio	Genes
leukemia	4/78	0.066	9.86	BCL2;IKZF1;LPP;RUNX1
lymphoma	2/22	0.260	17.48	MYC;BCL2
cdeficiency	1/13	0.843	14.79	CD8A
rheumatoid arthritis	1/13	0.983	14.79	RUNX1
gastric cancer	1/11	1.000	17.48	CDH1
dementia	1/12	1.000	16.03	APP
ovarian cancer	1/13	1.000	14.79	CDH1
orofacial cleft	1/19	1.000	10.12	CDH1
alzheimer disease	1/26	1.000	7.40	APP
breast cancer	1/28	1.000	6.87	CDH1

Healthy				
Term	Overlap	Adjusted P-value	Odds Ratio	Genes
spinocerebellar ataxia	3/37	1.000	4.63	ATXN1;FGF14;PPP2R2B
cone-rod dystrophy	2/15	1.000	7.62	RIMS1;PITPNM3
cataract	3/47	1.000	3.65	SIX6;SORD;CHMP4B
ataxia	3/60	1.000	2.86	ATXN1;FGF14;PPP2R2B
colorectal cancer	2/40	1.000	2.86	MSH2;BRAF
leukemia	3/78	1.000	2.20	MYB;LMO2;ARHGAP26
cardiomyopathy	2/42	1.000	2.72	DES;MYL3
myopathy	2/44	1.000	2.60	DES;BIN1
long qt syndrome	1/12	1.000	4.76	CACNA1C
arrhythmogenic right ventricular dysplasia	1/13	1.000	4.40	RYR2

C. DisGeNET disease

Tumour unique

Term	Overlap	Adjusted P-value	Odds Ratio	Genes
Leukemogenesis	20/730	7.99E-06	5.32	APP; IL15; NCOA3; ARID5B; PTPRJ; IKZF1; CXXC5; LPP; CBFA2T3; AICDA; FLI1; RUNX1; AHI1; CDK6; SELL; CDH1; CD8A; MYC; BCL2; TP73
Precursor Cell Lymphoblastic Leukemia Lymphoma	16/618	5.20E-04	5.027	IL15; UHRF1; ARID5B; NFATC2; PTPRJ; IKZF1; CXXC5; CBFA2T3; AICDA; RUNX1; CDK6; CD8A; MYC; BCL2; CD47; TP73
Blood basophil count (lab test)	10/224	7.91E-04	8.669	AHI1; SPRED2; CDK6; BCL2; NFATC2; IKZF1; LPP; EXOC2; RUNX1; UBAC2
Eosinophil count result	9/201	0.0016	8.694	SPRED2; CDK6; BCL2; NFATC2; IKZF1; LPP; EXOC2; RUNX1; UBAC2
Eosinophil count procedure	9/201	0.0019	8.694	SPRED2; CDK6; BCL2; NFATC2; IKZF1; LPP; EXOC2; RUNX1; UBAC2
Leukemia, Myeloid, Chronic-Phase	6/71	0.0019	16.41	MYC; RASA1; IRF2; IKZF1; RUNX1; TP73
Carcinogenesis	42/4065	0.0020	2.006	APP; RNASEH2B; TNFAIP8; SATB1; UHRF1; SDC2; ASAP3; CEND1; PTPRJ; IKZF1; CDH8; FAM107B; RASSF2; RNF216; CDH1; MYC; IL6R; ZBTB7A; MBNL1; IL15; NCOA3; HUWE1; NFATC2; CBFA2T3; AICDA; FLI1; RUNX1; TIAM2; AHI1; CDK6; TRAF4; RASA1; KIF26B; PLA2G10; IRF2; KCNMA1; BCL2; TCF4; CD47; LGR5; EXOC2; TP73
Diffuse Large B-Cell Lymphoma	14/575	0.0021	4.728	APP; PTPRJ; IKZF1; LPP; AICDA; FLI1; SELL; CDH1; MYC; IRF2; PLCG2; BCL2; EXOC2; TP73
Lupus Erythematosus, Systemic	20/1113	0.0021	3.489	API5; IL15; ARID5B; NFATC2; IKZF1; LPP; AICDA; FLI1; RUNX1; RASGRP3; AHI1; SPRED2; CDK6; SELL; CD8A; RASA1; IRF2; BCL2; S1PR1; IL6R
Lymphoma	21/1307	0.0026	3.12	IL15; SATB1; HUWE1; NFATC2; PTPRJ; IKZF1; CBFA2T3; AICDA; FLI1; RUNX1; AHI1; CDK6; CDH1; CD8A; MYC; PLCG2; BCL2; GPC5; CD47; ZBTB7A; TP73
Acute lymphocytic leukemia	17/907	0.0033	3.639	IL15; UHRF1; ARID5B; NFATC2; IKZF1; CXXC5; CBFA2T3; AICDA; FLI1; RUNX1; CDK6; MYC; BCL2; TCF4; CD47; ZBTB7A; TP73
leukemia	26/1941	0.0033	2.601	APP; UHRF1; HDC; IKZF1; CDH1; MYC; IL6R; ZBTB7A; IL15; NCOA3; ARID5B; NFATC2; CBFA2T3; AICDA; FLI1; RUNX1; AHI1; CDK6; SELL; CD8A; RASA1; IRF2; BCL2; TCF4; CD47; TP73
Leukemia, Myelocytic, Acute	24/1703	0.0033	2.736	APP; TRIO; IL15; SATB1; ARID5B; PTPRJ; IKZF1; CXXC5; DEXI; LPP; CBFA2T3; FLI1; RUNX1; CDK6; SELL; CDH1; MYC; RASA1; IRF2; BCL2; CD47; IL6R; ZBTB7A; TP73
Leukemia, Lymphocytic, Acute, L2	5/53	0.0054	18.32	CDK6; MYC; ARID5B; IKZF1; RUNX1
Squamous cell carcinoma of skin	8/205	0.0057	7.578	CDK6; MYC; RASA1; BCL2; LPP; LGR5; AICDA; TP73
Colorectal Cancer	35/3298	0.0060	2.061	APP; TRIO; SATB1; UHRF1; SDC2; HDC; ASAP3; PTPRJ; IKZF1; JAKMIP1; LPP; RASSF2; CDH1; ADORA3; MYC; FAM110B; IL6R; MCF2L2; MBNL1; IL15; NCOA3; HUWE1; CBFA2T3; AICDA; RUNX1; CD8A; RASA1; KIF26B; PLA2G10; BCL2; TCF4; ALPK2; CD47; LGR5; TP73
Metastatic melanoma	11/417	0.0062	5.122	APP; TRIO; CDK6; IL15; MYC; HDC; BCL2; NFATC2; ALPK2; CD47; TP73
Malignant neoplasm of lung	29/2449	0.0063	2.299	APP; TNFAIP8; SATB1; UHRF1; ASAP3; LPP; RASSF2; CDH1; ADORA3; MYC; GPC5; IL6R; ZBTB7A; IQSEC1; IL15; NCOA3; HUWE1; NFATC2; AICDA; CDK6; TRAF4; RASA1; PLA2G10; IRF2; BCL2; COL4A5; TCF4; CD47; TP73
Myeloid Leukemia, Chronic	16/876	0.0066	3.547	IL15; HDC; IKZF1; AICDA; RUNX1; AHI1; SPRED2; CDK6; SELL; CDH1; MYC; RASA1; IRF2; BCL2; TCF4; TP73
Blast Phase	8/214	0.0067	7.259	CDK6; CDH1; MYC; BCL2; IKZF1; AICDA; RUNX1; TP73
Lymphoid leukemia	8/213	0.0068	7.293	IL15; UHRF1; MYC; BCL2; IKZF1; AICDA; RUNX1; TP73
Primary Effusion Lymphoma	7/153	0.0070	8.884	CDK6; MYC; BCL2; IKZF1; CD47; IL6R; FAM107B

Low grade glioma	5/62	0.0072	15.66	CDH1; SATB1; MYC; TCF4; TP73
B-Cell Lymphomas	14/730	0.0085	3.724	TRIO; IL15; HUWE1; PTPRJ; DEXI; CBFA2T3; AICDA; RUNX1; AHI1; CDK6; CDH1; MYC; BCL2; TP73
Primary malignant neoplasm of lung	27/2268	0.0086	2.312	APP; TNFAIP8; SATB1; ASAP3; LPP; RASSF2; CDH1; ADORA3; MYC; GPC5; IL6R; ZBTB7A; IL15; NCOA3; HUWE1; NFATC2; AICDA; CDK6; TRAF4; RASA1; PLA2G10; IRF2; BCL2; COL4A5; TCF4; CD47; TP73
Breast Carcinoma	45/4963	0.0087	1.761	APP; TNFAIP8; TRIO; SH3KBP1; SATB1; UHRF1; SDC2; HDC; ASAP3; PTPRJ; CXXC5; LPP; FAM107B; RASGRP3; RASSF2; CDH1; MYC; PLCG2; S1PR1; IL6R; ZBTB7A; API5; IQSEC1; IL15; NCOA3; KRT13; NFATC2; FRMD4A; CBFA2T3; AICDA; FLI1; RUNX1; CDK6; TRAF4; DAD1; RASA1; KIF26B; PLA2G10; KCNMA1; BCL2; TCF4; CD47; ASTN2; LGR5; TP73
Solid Neoplasm	15/840	0.0093	3.467	IL15; SDC2; NFATC2; CXXC5; AICDA; FLI1; RUNX1; CDH1; CD8A; MYC; BCL2; S1PR1; CD47; ZBTB7A; TP73
Richter's syndrome	4/34	0.0096	22.84	MYC; BCL2; LRP4; AICDA
Acute Myeloid Leukemia, M1	6/116	0.0102	10.04	TRIO; CDK6; MYC; BCL2; ZBTB7A; RUNX1
Acute Myeloid Leukemia (AML-M2)	6/121	0.0108	9.629	TRIO; CDK6; MYC; BCL2; ZBTB7A; RUNX1
Chondrosarcoma	8/241	0.0108	6.446	TRAF4; MYC; SDC2; HDC; BCL2; COL4A5; NFATC2; ZBTB7A
Childhood B Acute Lymphoblastic Leukemia	3/13	0.0109	44.81	ARID5B; IKZF1; RUNX1
Marginal Zone B-Cell Lymphoma	5/74	0.0109	13.12	CDH1; MYC; BCL2; PTPRJ; AICDA
Carcinoma of lung	28/2476	0.0109	2.196	APP; TNFAIP8; SATB1; UHRF1; ASAP3; LPP; RASSF2; CDH1; ADORA3; MYC; GPC5; IL6R; ZBTB7A; IL15; NCOA3; HUWE1; NFATC2; AICDA; CDK6; TRAF4; RASA1; PLA2G10; IRF2; BCL2; COL4A5; TCF4; CD47; TP73
Familial primary gastric lymphoma	5/72	0.0111	13.48	SELL; CDH1; MYC; BCL2; AICDA
Gastritis	7/181	0.0112	7.51	SELL; CDH1; IL15; BCL2; LGR5; AICDA; TP73
Colonic Neoplasms	14/778	0.0113	3.494	APP; ACY1; SH3KBP1; IL15; UHRF1; NCOA3; NFATC2; RUNX1; CDH1; ADORA3; MYC; BCL2; TCF4; LGR5
Malignant neoplasm of breast	45/5054	0.0113	1.729	APP; TNFAIP8; TRIO; ACY1; SH3KBP1; SATB1; UHRF1; SDC2; HDC; PTPRJ; CXXC5; LPP; FAM107B; RASGRP3; RASSF2; CDH1; MYC; PLCG2; S1PR1; IL6R; ZBTB7A; MCF2L2; API5; IQSEC1; IL15; NCOA3; KRT13; NFATC2; CBFA2T3; AICDA; FLI1; RUNX1; CDK6; TRAF4; CD8A; RASA1; KIF26B; PLA2G10; KCNMA1; BCL2; TCF4; CD47; ASTN2; LGR5; TP73
Colorectal Carcinoma	31/2931	0.0114	2.054	APP; TRIO; SATB1; UHRF1; SDC2; ASAP3; PTPRJ; IKZF1; LPP; RASSF2; CDH1; MYC; IL6R; ZBTB7A; MBNL1; IL15; NCOA3; HUWE1; NFATC2; AICDA; RUNX1; CD8A; RASA1; KIF26B; PLA2G10; BCL2; TCF4; ALPK2; CD47; LGR5; TP73
Autistic Disorder	13/677	0.0115	3.729	APP; ACY1; IL15; SDC2; JAKMIP1; CDH8; FAM107B; AHI1; SYNGAP1; KCNMA1; BCL2; TCF4; ASTN2
Hodgkin Disease	12/592	0.0128	3.936	AHI1; SELL; SATB1; MYC; BCL2; NFATC2; IL6R; DEXI; LPP; CBFA2T3; AICDA; TP73
Chronic Lymphocytic Leukemia	17/1120	0.0130	2.947	IL15; SDC2; LRP4; LPP; AICDA; SELL; CDH1; CD8A; MYC; IRF2; PLCG2; BCL2; S1PR1; CD47; IL6R; ZBTB7A; TP73
Liver carcinoma	35/3593	0.0161	1.891	APP; TNFAIP8; TRIO; SATB1; UHRF1; SDC2; ASAP3; RASGRP3; SPRED2; CDH1; ADORA3; MYC; S1PR1; GPC5; IL6R; ZBTB7A; API5; IQSEC1; IL15; NCOA3; HUWE1; NFATC2; AICDA; TIAM2; AHI1; CDK6; RASA1; PLA2G10; IRF2; BCL2; TCF4; ALPK2; CD47; LGR5; TP73
Primary malignant neoplasm	16/1032	0.0164	3.01	API5; IL15; SATB1; UHRF1; SDC2; NCOA3; FLI1; FAM107B; RASGRP3; TRAF4; CDH1; MYC; KIF26B; BCL2; CD47; LGR5

Healthy				
**Term**	**Overlap**	**Adjusted P-value**	**Odds Ratio**	**Genes**
Body mass index	25/370	0.0034	3.21	SCARB2; SEMA5B; GBE1; ATP10A; FHIT; NPAS3; SNX29; LIPC; ADAMTS17; FAM19A2; RSPO3; MACROD2; CTNNBL1; LINGO2; KDM4C; COL25A1; LMNTD1; ITPK1; GAB2; SMAD6; LRP1B; MMS22L; ZFAT; CDH12; LMX1B
Finding of body mass index	25/370	0.0017	3.21	SCARB2; SEMA5B; GBE1; ATP10A; FHIT; NPAS3; SNX29; LIPC; ADAMTS17; FAM19A2; RSPO3; MACROD2; CTNNBL1; LINGO2; KDM4C; COL25A1; LMNTD1; ITPK1; GAB2; SMAD6; LRP1B; MMS22L; ZFAT; CDH12; LMX1B
Drug habituation	14/138	0.0044	4.819	TMPRSS7; SAMD4A; XYLT1; GRIK2; NR3C1; FHIT; THSD4; TIAM2; RAD51B; MSRA; CHCHD3; POGZ; CTNNA3; MACROD2
Drug Use Disorders	13/119	0.0034	5.19	TMPRSS7; SAMD4A; XYLT1; GRIK2; FHIT; THSD4; TIAM2; RAD51B; MSRA; CHCHD3; CTNNA3; MACROD2; CTNNBL1
Substance Dependence	14/140	0.0031	4.751	TMPRSS7; COL25A1; SAMD4A; XYLT1; GRIK2; FHIT; THSD4; TIAM2; RAD51B; MSRA; CHCHD3; CTNNA3; MACROD2; PKNOX2
Drug Dependence	15/175	0.0077	4.072	TMPRSS7; RASGRF2; SAMD4A; XYLT1; BRAF; GRIK2; FHIT; THSD4; TIAM2; RAD51B; MSRA; CHCHD3; CTNNA3; MACROD2; PKNOX2
Organic Mental Disorders, Substance-Induced	12/116	0.0087	4.914	TIAM2; RAD51B; MSRA; TMPRSS7; CHCHD3; SAMD4A; XYLT1; CTNNA3; MACROD2; GRIK2; FHIT; THSD4
Prescription Drug Abuse	12/116	0.0076	4.914	TIAM2; RAD51B; MSRA; TMPRSS7; CHCHD3; SAMD4A; XYLT1; CTNNA3; MACROD2; GRIK2; FHIT; THSD4
Substance Use Disorders	13/143	0.0116	4.319	CNTNAP2; TMPRSS7; SAMD4A; XYLT1; GRIK2; FHIT; THSD4; TIAM2; RAD51B; MSRA; CHCHD3; CTNNA3; MACROD2
Substance-Related Disorders	12/123	0.0111	4.635	TIAM2; RAD51B; MSRA; TMPRSS7; CHCHD3; SAMD4A; XYLT1; CTNNA3; MACROD2; GRIK2; FHIT; THSD4
Unipolar Depression	27/517	0.0138	2.481	CHRM3; CNTNAP2; ITGB5; TENM4; MYT1L; ADAR; CACNA1C; NR3C1; FHIT; NPAS3; ADAMTS16; PDE11A; RELN; TDO2; MORC1; EPHB1; LHPP; CBX5; DST; GCH1; EGF; LSAMP; DCLK2; MRPL48; LMX1B; MCTP2; PDE9A
Calcification of coronary artery	22/375	0.0136	2.787	SHC4; RFC5; CNTNAP2; ST6GALNAC2; NEGR1; SLC39A11; PKD2L1; WDR72; RAB3IL1; ABCB11; MTSS1; LRP1B; RALYL; LIPC; NKAIN3; TBL2; ATXN1; RSPO3; PDSS2; GPC5; MORC1; SLC37A3
Abnormal behavior	21/372	0.0342	2.682	HSPA9; MPST; CNTNAP2; LINGO1; MBNL1; GBE1; CELF4; PRKCA; GRIK2; ADRB2; CACNA1C; NR3C1; NPAS3; AHI1; GCLC; RELN; TDO2; ERBB4; PPP2R2B; SMPD1; PIP4K2A
Forced expiratory volume function	10/101	0.0376	4.704	CHRM3; SNTG2; CFDP1; IREB2; CELF4; COBL; SPOCK1; KPNA4; ADRB2; THSD4
Waist-Hip Ratio	11/122	0.0358	4.283	PCSK2; CDH4; TENM4; GCH1; GREB1; FAM19A2; RSPO3; GPC5; MACROD2; SMAD6; GPC6
High density lipoprotein measurement	19/324	0.0385	2.786	CUBN; SEMA5B; GCH1; ITPK1; CACNA1C; NR3C1; NUP93; SMPD3; MSRA; CCND3; LIPC; TBL2; TMEM117; CTH; IL1RAPL1; TIMP3; RSPO3; SRGAP2; MYO1H

Supplementary Table 6| Gene enrichment analysis of genes closest to IS. EnrichR[10,11] was used for gene enrichment analysis. We looked at **a,** KEGG pathways[12], **b,** disease genes from OMIM (https://omim.org/) and **c,** DisGeNET[13]. We found significant enrichments related to cancer and leukaemia in the tumour unique data set. “Overlap” gives the number of genes in our dataset that are present in the database category compared to the total number of genes in that category. Only top 10 genes are shown if there are no further significant hits. *P*-values from *Enrichr* are calculated using Fisher's Exact Test and adjusted for multiple testing.

Dataset (ref)	Tumour-specific IS			Healthy IS (koala unique)			Total genes in dataset
	Number of genes (total=106)	Enrichment	*P*	Number of genes (total=366)	Enrichment	*P*	
Bushman cancer	26	1.66	0.0051	66	1.22	0.0869	2266
Harminizome Lymphoma	20	2.94	2.28E-07	34	1.45	0.0279	983
Intogen	7	2.38	0.0174	13	1.28	0.3745	425
DriverDB	18	1.42	0.1156	71	1.62	1.84E-05	1835
Cancer Genome Atlas	11	2.03	0.0148	40	2.13	8.80E-07	784

Supplementary Table 7| Gene enrichments in cancer databases for sets of genes closest to IS. Five cancer gene data sets were used to test for enrichments in our gene sets. Total number of annotated koala genes = 15301. Significance calculated using a two-tailed 2 x 2 *Chi square* test.

	Gene	Dist		cov	Protein function
**Kathy**	*CUNH9orf3*	0	+	43	Orthologous to AOPEP (C9ORF3)
	SDC2	0	+	33	Altered syndecan-2 expression has been detected in several different tumor types
	HDC	-2638	-	32	Converts L-histidine to histamine
	KIF26B	0	+	32	KIF26B, a novel oncogene, promotes proliferation and metastasis by activating the VEGF pathway in gastric cancer. High expression of KIF26B in breast cancer associates with poor prognosis
	RASSF2	0	-	32	Potential tumor suppressor. Acts as a KRAS-specific effector protein.
	CD8A	7032	-	32	
	RNF216	0	-	29	RNF216 contributes to proliferation and migration of colorectal cancer via suppressing BECN1-dependent autophagy.
	RASA1	-168374	+	27	Suppressor of RAS function allowing control of cellular proliferation and differentiation.
	LIN52	0	-	27	Among its related pathways are Cell Cycle, Mitotic and Cellular senescence
	CXXC5	-37933	-	27	Involved in myelopoiesis, is required for DNA damage-induced p53 activation,
**Lisa**	*LOC110213472*	0	-	107	
	ZBTB7A	0	-	103	ZBTB7A acts as a tumor suppressor through the transcriptional repression of glycolysis
	CUNH1orf21	0	+	96	
	BCL2	0	-	95	B-cell lymphoma 2 blocks the apoptotic death of some cells such as lymphocytes
	LOC110211318	-169704	-	94	
	SCIMP	0	-	92	Transmembrane adaptor protein that is expressed in antigen-presenting cells and is localized in the immunologic synapse.
	LPP	-222740	+	91	
	LOC110205351	-1165	-	87	
	LOC110202304	0	-	86	
	LOC110210622	5653	-	82	
**Ralf**	*SPRED2*	-35875	+	36	
	TRIO	0	-	28	GDP to GTP exchange factor. This protein promotes the reorganization of the actin cytoskeleton, thereby playing a role in cell migration and growth. Upregulated TRIO expression correlates with a malignant phenotype in human hepatocellular carcinoma.
	ADORA3	11317	-	16	
	LOC110208445	0	-	14	
	LOC110211725	-605	+	14	
	CUNH10orf99	-10627	+	13	
	PPP1R1C	0	-	13	
	FAM110B	-297232	+	12	
	TIAM2	0	+	11	T Cell Lymphoma Invasion And Metastasis 2.
	NFATC2	0	-	11	Nuclear Factor Of Activated T Cells 2. Tumor suppressor in untransformed cells, but may act as an oncogene in cancer cells.
**A31756**	*RASA4B*	0	-	92	Ras GTPase-Activating Protein 4B
(No tissue)	*RUNX1*	0	+	90	Involved in the generation of hematopoietic stem cells and for their differentiation into myeloid and lymphoid lines. Loss of RUNX1 function has been shown to impair differentiation between myeloid and lymphoid lines often resulting in the development of leukemia.
	TP73	0	-	89	Tumor Protein P73. Participates in the apoptotic response to DNA damage. May be a tumor suppressor protein.
	GTDC1	-48063	-	85	
	LOC110218567	-39815	-	82	
	API5	-143685	+	75	
	GTDC1	-25455	-	72	
	PTPRJ	0	+	72	
	ARID5B	-20557	-	70	
	LOC110213370	0	+	67	

Supplementary Table 8| Top tumour-specific IS in four koalas (by coverage) with over 20 tumour-specific IS. Genes tested by qPCR are highlighted; these were all named genes (not unannotated transcripts) which contained an intronic IS and had high coverage. No liver or lymph node tissue was available for koala A31756 for qPCR.

Liver samples	Mirali	Ralf	Buster	Butler	Kathy	Lisa	Bilyarra	*t test*	*P*
RASSF2	-16.21	-17.36	-17.54	-18.95	-12.19	NA	-15.63	7.69	**0.0023**
RNF216	-17.08	-18.79	-20.93	-20.14	-14.62	NA	-16.88	4.60	**0.0096**
SDC2	-10.68	-12.59	-10.56	-10.89	-9.36	NA	-9.15	2.31	0.0521
KIF26B	-17.83	-19.58	-15.47	-18.89	-8.78	NA	-15.38	8.95	**0.0015**
BCL2L1 (fusion)	-15.34	-15.84	-15.28	-15.14	-2.26	NA	-11.37	13.54	**0.0004**
**Lymph node samples**									
TRIO	-17.86	-14.77	NA	NA	NA	-20.01	-20.98	4.29	0.0729
TIAM2	-13.27	-21.92	NA	NA	NA	-15.73	-13.00	-7.45	**0.0425**
BCL2	-23.52	-19.16	NA	NA	NA	-18.5	-23.66	2.00	0.1478

Supplementary Table 9| qPCR results from healthy and tumour tissues (ΔCT values). Orange = cancer tissue with integration site in gene being tested; blue = cancer tissue but no integration site near gene; no highlight = healthy tissue. Significance was calculated on ΔCT values using a one-sided *t*-test. Livers and lymph nodes from two koalas from outside this study (Bilyarra and Mirali) were used as extra controls. Bilyarra was used to calculate ΔΔCT for each koala.

Samples used:

Liver:

- Kathy = tumour tissue with IS in gene being tested
- Ralf = tumour tissue without IS
- Buster and Butler = healthy liver
- Mirali and Bilyarra = additional controls

Lymph node:

- Ralf = tumour tissue with IS in *TRIO* and *TIAM2*
- Lisa = tumour tissue with IS in *BCL2*
- Mirali and Bilyarra = additional controls

Koala group	Gene	Position of IS	Gene significance
Donovan/Bonnie/Butler	***LSAMP***	**3' UTR**	Candidate tumor suppressor gene in human osteosarcomas
	CCDC25	Intron	High expression of CCDC25 in cholangiocarcinoma tissue samples
	LMF1	Intron	
	PCM1	-1651	Chromosomal aberrations associated with papillary thyroid carcinomas and a variety of hematological malignancies
	SCNN1B	-26778	
	MMS22L	51653	Overexpressed in most lung and esophageal cancers.
Elise/Ralf	***ZFAT***	**5' UTR (alt trans.)**	Constitutive expression thought to induce tumor growth and angiogenesis
	HMBOX1	**3' UTR**	Overexpression in hematopoietic proginators or T-cells may contribute to oncogenic transformation
	FSD2	Intron	
	MGAT4C	Intron	Significantly over-expressed in metastatic versus localized prostate cancer
	PDE9A	Intron	Breakpoints in PDE9A associated with lymphoblastic leukaemia
	CCDC90B	-2890	
	PLB1	-9354	
	SAV1	-12456	
	ADAR	14443	Promotes malignant progenitor reprogramming in chronic myeloid leukemia.
	PARD3B	135847	
Kathy/A30038	***TMEM65***	**5' UTR alt trans**	
	MTSS1	**3 bases 5' of start of alt trans.**	Tumor suppressor in chronic myeloid leukaemia
	CELF4	Intron	
	SLC37A3	Intron	
	NAPG	3456	
	MYO1H	5274	
	NOA1	-5987	
Lisa/A31756	*DCDC2C*	Intron	
	GCH1	Intron	
	NKAIN3	Intron	
	NOMO2	Intron	
	TENM4	Intron	Tumor suppressor; mutations identified in ~ 50% of primary lymphomas of the CNS
	SCAPER	-1622	
	ZBED3	7887	Upregulated in lung cancer tissues
	CLYBL	-63808	
	BNIP2	172271	
	LITAF	-46729	

Supplementary Table 10| Koala groups containing shared IS in gene regions. Only IS in or closest to annotated genes are shown. Position of IS in transcript is given or distance to nearest gene. Genes in bold have IS that are likely to interfere with the gene transcript. "Alt trans" = Gene has alternative transcripts with different start positions.

Chr	Region size	Number of IS	Random occurrence	Adjusted Poisson P value	Nearby Genes	Gene biotypes	Info	Cancer?	Body weight/other
NW_018344101.1: 3616809-3787615	170806	8<br><br>[7 ERV, 1 TS]	0	2.77E-11	***CLYBL***<br>***TM9SF2***<br>*LOC110192671*<br>*LOC110192670*<br>*LOC11019269*<br>*ZIC5*	protein<br>protein<br>lncRNA<br>lncRNA<br>protein<br>protein	5' of *CLYBL*	*TM9SF2*	*CLYBL*: vitamin B12 metabolism
NW_018344041.1: 3837741-3959328	121587	7<br><br>[1 ERV, 3 TS, 3 HS]	0	5.51E-10	*LOC110218567*<br>*LOC110218555*<br>*LOC110218554*<br>*LOC110218475*<br>*TRNAI-UAU*<br>***HAAO***	ncRNA<br>lncRNA<br>lncRNA<br>lncRNA<br>tRNA<br>protein	*Mostly tissue-specific IS. This is an unusual region with many low coverage IS*		*HAAO*: kynurine pathway
NW_018344261.1: 742388-829740	87352	6<br><br>[6 ERV]	0	1.98E-08	***PPFIBP1***<br>*LOC110201477*<br>*CUNH12orf71*<br>*REP15*<br>*MRPS35*<br>*SMCO2*	protein<br>lncRNA<br>protein<br>protein<br>protein<br>protein	intron of *PPFIBP1*,<br>5' of lncRNA *LOC110201477*	*PPFIBP1*	*PPFIBP1*
NW_018343993.1: 9691043-9856264	165221	5<br><br>[5 ERV]	0.0007	5.45E-05	***SPOCK1***<br>*LOC110211746*<br>*KLHL3*<br>*HNRNPA0*<br>*LOC110211809*<br>*LOC110211807*	protein<br>lncRNA<br>protein<br>protein<br>lncRNA<br>protein	5' and 1st intron of *SPOCK1*	*SPOCK1*	*SPOCK1*
NW_018343979.1: 18435185-18618425	183240	5<br><br>[5 ERV]	0.0007	8.21E-05	***AHI1***<br>*LOC110208912*<br>***MYB***<br>*HBS1L*<br>*ALDH8A1*<br>*PDE7B*	protein<br>lncRNA<br>protein<br>protein<br>protein<br>protein	intron of *AHI1*	*AHI1*	*AHI1*
NW_018344021.1: 11254169-11254793	624	3<br><br>[2 ERV, 1 TS]		6.21E-06	***MYC***<br>*LOC110216080*<br>*LOC110216056*<br>*LOC110216057*<br>*LOC110216109*<br>*LOC110216110*	protein<br>lncRNA<br>lncRNA<br>lncRNA<br>lncRNA<br>lncRNA	5' of *MYC*	*MYC*	
NW_018344239.1: 52532-54214	1682	3<br><br>[3 ERV]	0.0003	4.51E-05	***LOC110200921***<br>*DIO2*<br>*LOC110200915*<br>*CEP128*	protein<br>protein<br>lncRNA<br>protein	At very end of contig so 5' genes unknown; *LOC110200921 = NRXN3-like*		(*NRXN3*)

Supplementary Table 11| Regions of statistically high IS density (hotspots). Random occurrence indicates the number of times that this number of IS was seen in a window of this size in 10,000 Monte Carlo simulations. The Poisson *P* value (adjusted for number of windows tested) is also given. The region containing *HAAO* also contains the most significantly dense region of tumour-specific IS in the genome; this is an usual region compared to the others as it contains a high number of tissue-specific (somatic) IS. The number of IS in each hotspot is given along with information on the types of IS: ERV = IS in both tissues, TS = tumour-specific,

HS = healthy-specific. Nearby genes that have cancer associations are indicated, as are genes involved in body weight or other important metabolic pathways.

Experiment	Primer name	Direction	Sequence
SIP and IS confirmation	KoRV LTR F	fwd	ATT TGC ATC CGG AGT TGT GT
	KoRV LTR R	rev	AGG GGC ACC CTA GAA ACT GT
IS confirmation (tumour-specific IS)	TCF4 F	fwd	ACA GGC ACA AGT GGA TAT GGG
	TCF4 R	rev	TAC ACA AAC CTC TGG GGA CC
	RASSF2 F	fwd	TCA AAA ACA GAG CCT TGC AAC T
	RASSF2 R	rev	GGG AAG GAC TTG TTT GCC TG
	CDK6 F	fwd	CGG GCT GCC CAG AGA TAT G
	CDK6 R	rev	TAG GTT CAT GCC TCG CTT CTG
	RAD51B F	fwd	CGA GGC AAG AAC TGT AGG GG
	RAD51B R	rev	ATG CCT TCA CAT AGT AGG GGC
	SCIMP F	fwd	GGG TTT CCC TGC CCA GAT AC
	SCIMP R	rev	AAG GGA GAC CCT GAA ATG CC
	RNF216 F	fwd	GCC TCC AAA TGA CTG CCC TAA
	RNF216 R	rev	TCC ATT TGC ATC CTA GCC TCC
	TRIO F	fwd	GAG GAA GGG AGG AGT TGG ATT C
	TRIO R	rev	TGC AGT AGG TTA AGG TTC AGA ATG
	ZBTB7A F	fwd	AGG AAC GAA CTA CAC GCT GG
	ZBTB7A R	rev	ATC AGA GAG AAG AAT GGT CTG GT
Gene Expression (qPCR)	RAD51B F	fwd	ACC ACT TGC TCC GTT CTC TG
	RAD51B R	rev	TGT CTT CTC TTT GAG TCA AGG TAT C
	RASSF2 F	fwd	CCC TGA TTG CCA GAA TCC TCC
	RASSF2 R	rev	GAG GAC CTG AGG CGT TGT CA
	RNF216 F	fwd	TAC CTC TGT CGA GCC TCC AT
	RNF216 R	rev	TCT TTC CAT AGG CCC TGA CAC
	SDC2 F	fwd	GCG GAG CTG ACT TCT GAT AAA GA
	SDC2 R	rev	GAA TTG TTC GGG ATG TTG GCA T
	KIF26B F	fwd	TCA GCA TCT GAA TCA CCC CT
	KIF26B R	rev	GGA GCA GAT GCG AAC CAT GA
	TRIO F	fwd	TGA GTT GTC TTT GCT TGG AGG A
	TRIO R	rev	GCT TAC CCT GGG CTA CGA TT
	TIAM2 F	fwd	GCA TCA GAT AGT GGT CCC CG
	TIAM2 R	rev	TGC ATA TAT TCA TAA GGA GCT GGA
	BCL2 F	fwd	CCT TAA GGG ATA GAC TTC CAG G
	BCL2 R	rev	GGA AGC AGA ATG GTG AAG GC
	BCL2L1 F	fwd	GTC TTG GTA GGA CGA ATC AC
	BCL2L1 R	rev	TCG TTC CCA TAA AGC TCC AC
BCL2L1 in Elise	ZBTB18	fwd	GTG GAA GGT GTT AGG TTG AA
	BCL2L1 R	rev	TCG TTC CCA TAA AGC TCC AC

Supplementary Table 12| Primers used in this study. For IS confirmation (to confirm IS are tumour unique) we used the each KoRV LTR primer with each gene-specific primer in order to capture both ends of the IS regardless of KoRV orientation (i.e. forward KoRV LTR with each forward gene specific primer; forward KoRV LTR with each reverse gene specific primer; reverse KoRV LTR with each forward gene specific primer; reverse KoRV LTR with each

reverse gene specific primer). Only two combinations of primers should produce bands for each gene. See Supplementary Fig. 4 for more details.

For qPCR gene expression analysis, primers cover exon boundaries in all genes with more than two exons. Here we used forward and reverse gene primers together (not with KoRV LTR primer as for IS confirmation)

Supplementary References

1 Alquezar-Planas D. E. *et al.* DNA Sonication Inverse PCR for Genome Scale Analysis of Uncharacterized Flanking Sequences. *Methods Ecology Evol* **12**, 182-195 (2020).

2 Silver, J. & Keerikatte, V. Novel use of polymerase chain reaction to amplify cellular DNA adjacent to an integrated provirus. *J Virol* **63**, 1924-1928 (1989).

3 Lober, U. *et al.* Degradation and remobilization of endogenous retroviruses by recombination during the earliest stages of a germ-line invasion. *Proc Natl Acad Sci USA* **115**, 8609-8614 (2018).

4 Robinson, J. T. *et al.* Integrative genomics viewer. *Nat Biotechnol* **29**, 24-26 (2011).

5 Sarker, N. *et al.* Identification of stable reference genes for quantitative PCR in koalas. *Sci Rep* **8**, 3364 (2018).

6 Eiden, M. V. A novel exogenous retrovirus isolated from koalas (Phascolarctos cinereus) with malignant neoplasias in a United States zoo. *Technical Reports of the Australian Museum, Online* **24**, 3-4 (2014).

7 Chappell, K. J. *et al.* Phylogenetic Diversity of Koala Retrovirus within a Wild Koala Population. *J Virol* **91**, e01820-16 (2017).

8 Hobbs, M. *et al.* Long-read genome sequence assembly provides insight into ongoing retroviral invasion of the koala germline. *Sci Rep* **7**, 15838 (2017).

9 Quigley, B. L. *et al.* Changes in Endogenous and Exogenous Koala Retrovirus Subtype Expression over Time Reflect Koala Health Outcomes. *J Virol* **93,** e00849-19 (2019).

10 Chen, E. Y. *et al.* Enrichr: interactive and collaborative HTML5 gene list enrichment analysis tool. *BMC Bioinformatics* **14**, 128 (2013).

11 Kuleshov, M. V. *et al.* Enrichr: a comprehensive gene set enrichment analysis web server 2016 update. *Nucleic Acids Res* **44**, W90-97 (2016).

12 Kanehisa, M. & Goto, S. KEGG: kyoto encyclopedia of genes and genomes. *Nucleic Acids Res* **28**, 27-30 (2000).

13 Pinero, J. *et al.* DisGeNET: a comprehensive platform integrating information on human disease-associated genes and variants. *Nucleic Acids Res* **45**, D833-D839 (2017).
