# Supplementary material for: Retroviral integrations contribute to elevated host cancer rates during germline invasion
Source: Nat Commun. 2021 Feb 26;12:1316. doi: 10.1038/s41467-021-21612-7 (PMC7910482; doi:10.1038/s41467-021-21612-7)
Supplement: Supplementary file 2 — Descriptions of Additional Supplementary Files [file 41467_2021_21612_MOESM2_ESM.pdf]

Descriptions of Additional Supplementary Files

Supplementary Data 1

Description: List of all integration sites detected.
